# RNA-Seq of Human Breast Ductal Carcinoma *In Situ* Models Reveals Aldehyde Dehydrogenase Isoform 5A1 as a Novel Potential Target

**DOI:** 10.1371/journal.pone.0050249

**Published:** 2012-12-06

**Authors:** Hitchintan Kaur, Shihong Mao, Quanwen Li, Mansoureh Sameni, Stephen A. Krawetz, Bonnie F. Sloane, Raymond R. Mattingly

**Affiliations:** 1 Department of Pharmacology, Wayne State University School of Medicine, Detroit, Michigan, United States of America; 2 Center for Molecular Medicine and Genetics, Department of Obstetrics and Gynecology, Wayne State University School of Medicine, Detroit, Michigan, United States of America; 3 Barbara Ann Karmanos Cancer Institute, Wayne State University, Detroit, Michigan, United States of America; The University of Arizona, United States of America

## Abstract

Breast ductal carcinoma *in situ* (DCIS) is being found in great numbers of women due to the widespread use of mammography. To increase knowledge of DCIS, we determined the expression changes that are common among three DCIS models (MCF10.DCIS, SUM102 and SUM225) compared to the MCF10A model of non-tumorigenic mammary epithelial cells in three dimensional (3D) overlay culture with reconstituted basement membrane (rBM). Extracted mRNA was subjected to 76 cycles of deep sequencing (RNA-Seq) using Illumina Genome Analyzer GAIIx. Analysis of RNA-Seq results showed 295 consistently differentially expressed transcripts in the DCIS models. These differentially expressed genes encode proteins that are associated with a number of signaling pathways such as integrin, fibroblast growth factor and TGFβ signaling, show association with cell-cell signaling, cell-cell adhesion and cell proliferation, and have a notable bias toward localization in the extracellular and plasma membrane compartments. RNA-Seq data was validated by quantitative real-time PCR of selected differentially expressed genes. Aldehyde dehydrogenase 5A1 (ALDH5A1) which is an enzyme that is involved in mitochondrial glutamate metabolism, was over-expressed in all three DCIS models at both the mRNA and protein levels. Disulfiram and valproic acid are known to inhibit ALDH5A1 and are safe for chronic use in humans for other disorders. Both of these drugs significantly inhibited net proliferation of the DCIS 3D rBM overlay models, but had minimal effect on MCF10A 3D rBM overlay models. These results suggest that ALDH5A1 may play an important role in DCIS and potentially serve as a novel molecular therapeutic target.

## Introduction

Ductal carcinoma *in situ* (DCIS) of the breast results from the proliferation and accumulation of atypical epithelial cells that remain restricted to the duct and is a non-obligate precursor to invasive breast cancer. Increases in mammographic screening have led to a shift of the stages of breast cancer at diagnosis from locoregional disease (stages II and III) to DCIS or local disease (stages 0 or I). As a result, DCIS, which used to be an uncommon finding, is now being diagnosed in more than 60,000 patients each year in the US [1]. Thus, DCIS now accounts for 20–45% of all newly detected cancers in females undergoing breast screening [2]. Virtually all women diagnosed with DCIS undergo at least surgical resection, and many of them are subjected to adjuvant radiation and anti-estrogen therapy. Following this aggressive treatment, 5–30% of these DCIS cases will locally recur, with half of these recurrences being to invasive ductal carcinoma (IDC) [1]. Women undergoing primary breast conserving surgery for DCIS with or without IDC have a higher re-operation rate than those with isolated invasive disease [3]. Re-operation is itself associated with further increased risk of subsequent local recurrence [4]. In addition to the problem of identifying additional therapeutic targets in these recurrent cases of DCIS, there is also the issue of over-treatment in the majority of DCIS cases that would remain indolent in the absence of surgery and radiation [5], [6]. Another challenge that DCIS presents is the heterogeneity of the lesions [7], [8]. Molecular profiling of DCIS samples indicates that all intrinsic subtypes that have been identified in invasive breast cancer are also recognized in DCIS [7]. The identification of therapeutic targets for DCIS should allow development of pre-surgical treatments to improve outcome in high risk patients [9], [10] as well as alternative strategies that do not have the side effects of hormone suppression. In addition preventive studies could be performed in women at lower risk because their DCIS is likely to remain indolent [11].

Pre-clinical therapeutic identification and development has mostly been based in conventional cell culture systems on plastic dishes. Cancer cells grown in three dimensional (3D) matrices, such as reconstituted basement membrane (rBM), have been proposed to exhibit responses and resistance to drugs that are closer to those observed *in vivo* [12], [13], [14]. To provide a tractable and relevant model for the investigation of DCIS, we have previously developed 3D rBM cultures of MCF10.DCIS cells [12], [15], [16]. MCF10.DCIS cells had previously been shown to initially form lesions characterized as comedo DCIS in immunodeficient mice; about 50% of the mature lesions later progress to invasive ductal carcinoma (IDC) [17]. We have extended the 3D rBM overlay culture model system to two additional DCIS cell lines derived from individual patients: SUM102 [18] and SUM225 [19]. SUM102 cells were isolated from a patient diagnosed with extensive ductal carcinoma *in situ* with areas of micro-invasion. SUM225 cells were derived from a chest wall recurrence in a patient previously diagnosed and treated for DCIS.

Over the past few years, tremendous technological developments in tissue micro-dissection and genomic technologies have enabled researchers to interrogate genetic changes that occur at the preinvasive stages of breast cancer. Several gene expression profiling studies of DCIS have been carried out using a combination of laser capture micro-dissection and microarrays [20], [21], [22], [23], [24], [25]. Serial analysis of gene expression found that the most dramatic transcriptome change occurs at the transition from normal epithelium to DCIS rather than from DCIS to invasive cancer [26]. This is supported by phenotypic and genomic analyses demonstrating that the molecular heterogeneity of breast ductal carcinomas is already established in *in situ* lesions [22], and studies from co-existing DCIS and IDC [23]. Increasing tumor grade and presence of necrosis have been associated with greater gene expression variability and distinct transcriptional signatures [20], [27]. Hannemann *et al.* identified a gene expression classifier of 35 genes that differed between DCIS and IDC and a panel of 43 genes which further distinguished well and poorly differentiated DCIS [28].

Although microarrays have been the technology of choice in most gene expression studies, they are limited by pre-defined probe sets and potential cross-hybridization. Next generation sequencing-based approaches like RNA-Seq do not have these limitations and offer unprecedented depth of analysis of gene expression. In the present study, we describe the first application of deep sequencing technology to identify differentially expressed transcripts and to explore the networks and pathways that underlie DCIS of the breast. A major goal of this approach was to identify new and potentially druggable targets [29]. We show that aldehyde dehydrogenase 5A1 (ALDH5A1) is over-expressed and that two independent drugs that inhibit its activity reduce net proliferation in DCIS 3D rBM overlay models.

## Materials and Methods

### Reagents

Disulfiram was a generous gift from Dr Angelika Burger (Karmanos Cancer Institute, Detroit, MI). Ham’s F-12 nutrient mixture (F-12), bovine serum albumin (BSA), hydrocortisone, dimethyl sulfoxide (DMSO), and valproic acid were purchased from Sigma (St. Louis, MO). Phosphate-buffered saline (PBS), horse serum, epithelial growth factor (EGF), insulin, and 3-(4, 5-dimethylthiazol-2-yl)-2,5-diphenyltetrazolium bromide (MTT) and Trizol® were purchased from Invitrogen (Carlsbad, CA). Mammary Epithelial Media 171 (M171) and Mammary Epithelial Growth Factor Supplement were from Cascade Biologics (Portland, OR). Fetal bovine serum (FBS) was from Hyclone (Logan, UT). Trypsin/EDTA solution, and penicillin-streptomycin were from Cellgro (Herndon, VA). Cultrex™ rBM was from Trevigen (Gaithersburg, MD). Total RNA prepared from human mammary epithelial cells was obtained from Cell Applications (San Diego, CA). Primary antibodies used for western blotting were mouse anti-GAPDH (EMD Chemicals, Darmstadt, Germany) and goat anti-ALDH5A1 (sc-70007, Santa Cruz Biotechnology, Santa Cruz, CA), with secondary antibodies and enhanced chemiluminescence detection as previously described [12].

### Cell Lines and Culture

MCF10A and MCF10.DCIS cell lines were obtained from the Cell Lines Resource (Karmanos Cancer Institute, Detroit, MI) and maintained as monolayers as previously described [12]. SUM102 and SUM225 cell lines were a generous gift from Dr. Stephen Ethier (Hollings Cancer Center, Charleston, SC) and were maintained as monolayers in Ham’s F-12 containing 5% fetal bovine serum, 5 µg/ml insulin and 1 µg/ml hydrocortisone, 50 U/ml penicillin, and 50 µg/ml streptomycin. The 3D rBM overlay culture system that we described previously [12], [15], [30] was modified to provide uniform culture conditions for all the cell lines by use of M171 media with Mammary Epithelial Growth Factor Supplement. Variants of MCF10.DCIS and SUM102 lines that express monomeric red fluorescent protein (mRFP) were developed by retroviral transduction as previously described [15].

### Harvest of 3D Structures

MCF10A, MCF10.DCIS, SUM102 and SUM225 cells were grown in 3D rBM overlay culture for 12 days with change of media every 4 days. Structures were harvested from rBM by repeated washes with ice-cold PBS supplemented with 5 mM EDTA.

### RNA Extraction and Purification

Total RNA was extracted using a combination of TRIZOL™ reagent and ethanol precipitation according to manufacturer’s instructions, with an additional purification step by on-column DNase treatment using the RNase-free DNase Kit (from Qiagen, Valencia, CA) to ensure elimination of any genomic DNA. The integrity and quantity of RNA in the samples was determined using NanoDrop 1000 spectrophotometer (Thermo Fisher Scientific, Waltham, MA) and Agilent 2100 Bioanalyzer (Agilent Technologies, Santa Clara, CA).

### Next Generation Sequencing

The libraries of template molecules for high throughput DNA sequencing were prepared using Illumina mRNA Sequencing Sample Preparation kit (Illumina, San Diego CA, USA). The library of products of desired size (150–200 bp) was then selected for further enrichment with 15 cycles of polymerase chain reaction (PCR) amplification. After validation of library using DNA 1000 chip (on Agilent Technologies 2100 Bioanalyzer), the samples were run on an Illumina Genome Analyzer GAIIx for 76 cycles of single-end sequencing. Image analysis and base calling were performed using the Firecrest and Bustard modules (Illumina Pipeline software v. 1.6.0). Sequencing reads were aligned to human reference genome (hg18). Alignments were performed with Novoalign (Novocraft Technologies SdnBhd, v. 2.05.43) using default parameters. Only unique alignments were considered for further analysis. The minimal number of reads per kilobase of exon model per million mapped reads (RPKM) to infer expression was 1. The next generation sequencing (NGS) analyzer from Genomatix (www.genomatix.de) was applied to cluster the alignments based on the distribution of aligned reads. NGS analyzer parameters were set as following: (1) the size of sliding window as 100 bp, (2) the minimum number of reads per cluster (τ) were calculated from the dataset applying a Poisson distribution. Two criteria were applied to determine differentially expressed transcripts between each of the DCIS models and MCF10A: |log_2_(fold change)| ≥2 and adjusted p-value <0.001. These values were calculated based on the default DESeq method [31] using the NGS analysis module “RegionMiner: Expression Analysis for RNA-Seq Data”. The sequence data from this study have been deposited in the National Center for Biotechnology Information’s (NCBI) Gene Expression Omnibus (GSE 36863).

### Pathway Analysis

Three different approaches were taken to find the molecular functions, gene ontology, and canonical pathways that were significantly associated with the differentially expressed genes in the three DCIS models. These approaches were: Ingenuity Pathway Analysis (IPA) (Redwood City, CA, USA); WEB-based GEne SeT AnaLysis Toolkit (WebGestalt); and Genomatix Pathway System (GePS). By default, P-value <0.05 was used as threshold to define enriched terms.

### Immunoblot Analysis

Cell lysates were prepared from the harvested 3D structures by addition of lysis buffer as described previously [12]. The lysates were briefly sonicated on ice, heated at 100°C for 5 minutes, separated by SDS-PAGE, transferred to nitrocellulose membranes for immunoblotting.

### Cell Viability and Proliferation Assays

The MTT dye reduction assay in 96-well microplates was used to determine cell viability. 1×10^4^ cells were plated in each well previously coated with Cultrex in a total volume of 200 µl of growth media. The wells were then treated with serial dilutions of drug and vehicle control for 3 days. After 3 days of drug treatment, MTT was added and further processed for absorbance as previously described [12]. After normalizing the absorbance values for blank and vehicle controls, the data were analyzed using GraphPad Prism version 5.0 by non-linear regression (curve fit) to plot sigmoid dose-response curves. The mRFP-expressing variants of MCF10.DCIS and SUM102 were grown in 3D rBM culture on coverslips with exposure to drug or vehicle controls for 8 days. To test for reversibility of growth inhibition, cultures were harvested after the 8-day treatment, and the cells re-plated in fresh growth media after dilution of the rBM. The cultures were continued in 2D without rBM for ten days in the absence of inhibitors and then cells were counted.

### Real-time Quantitative PCR Assay (qRT-PCR)

First-strand cDNA was synthesized from total RNA using High Capacity cDNA Reverse Transcription Kit (Applied Biosystems, Foster City, CA). The qRT-PCR reactions were carried out using diluted cDNA, 150 nM of each primer, and SYBR Green master mix (Applied Biosystems, Foster City, CA) in 20 µl reactions on a StepOnePlus™ Real-Time PCR System. Each sample was run in triplicate in separate wells for the target gene and three reference genes: hypoxanthine phosphoribosyltransferase 1 (HPRT1); β-actin (ACTB); and β-glucuronidase (GUSB). The average of three threshold cycle (Ct) values for the target and reference genes was used to determine the level of expression relative to the control. Delta-delta Ct method was used for data analysis. Primer pair sequences for all the genes are listed in the supplementary data (Table S1).

## Results

### Next Generation Sequencing of DCIS Models

**Figure 1:**
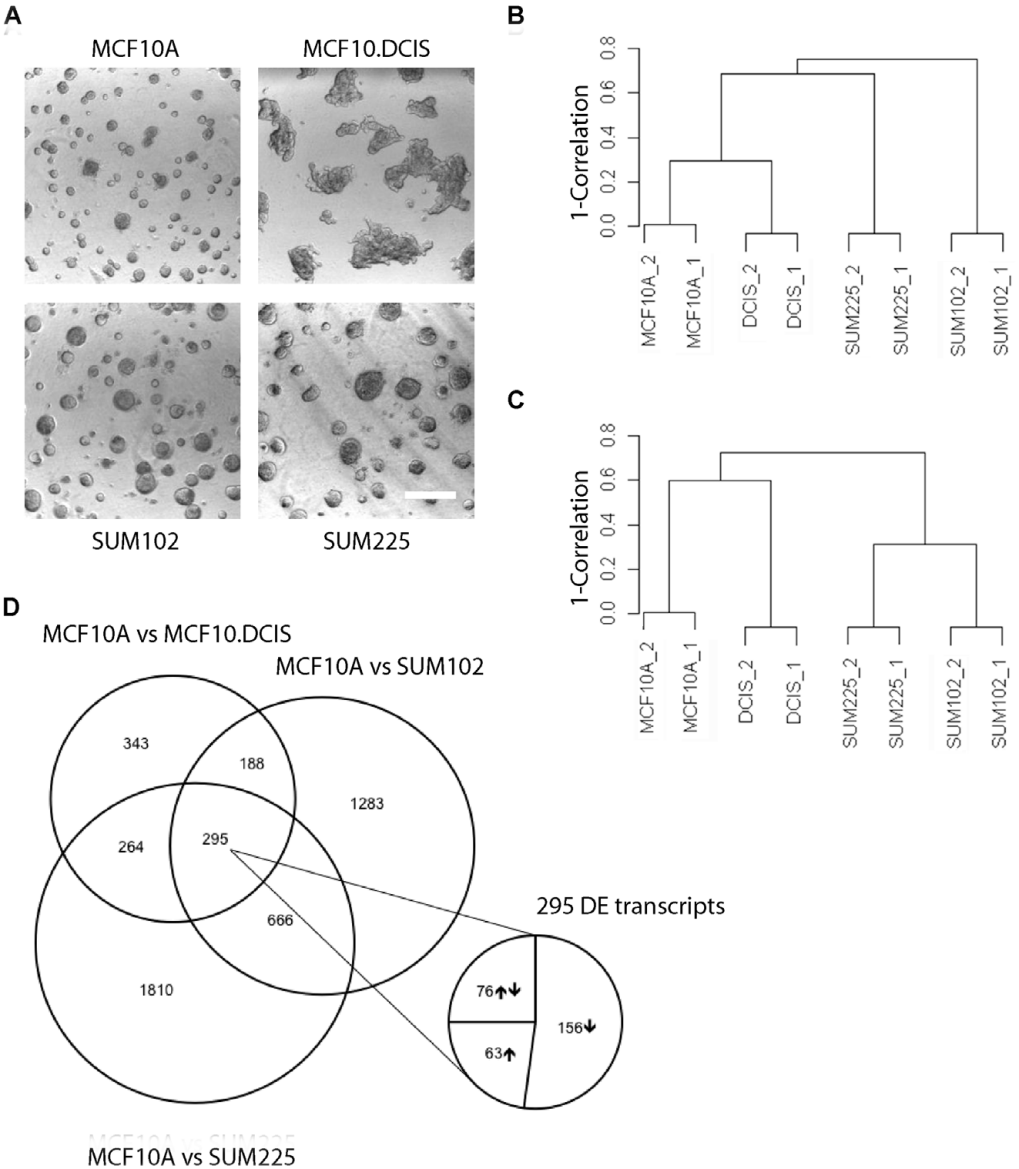
RNA-Seq analysis of MCF10A and DCIS models (*A*) Differential interference contrast (DIC) images of 12-day 3D rBM overlay cultures of MCF10A, MCF10.DCIS, SUM102, and SUM225 cells. Scale bar, 200 µm. (*B*) Unsupervised hierarchical clustering of the transcript profile from the eight RNA-Seq samples. (*C*) Cluster dendrogram of RNA-Seq samples based on differentially expressed genes. (*D*) Venn diagram of differential expression results (with a p-value <0.001 and cut-off fold change of 4) showing the overlap between the genes expressed by different models of DCIS in comparison to MCF10A. There are a total of 295 genes that are consistently differentially expressed between the MCF10A and the three DCIS models: MCF10.DCIS, SUM102 and SUM225. The expansion shows that 156 transcripts were decreased, 63 transcripts were increased, and 76 transcripts were differentially expressed in all three models but the direction of change was variable.

We compared 3D rBM overlay cultures of the three DCIS models (MCF10.DCIS, SUM102 and SUM225) to parallel cultures of non-tumorigenic MCF10A cells as a model for human mammary epithelial cells. All cultures were grown under uniform conditions with identical growth factors and supplements. After 12 days in 3D rBM overlay culture, the MCF10A cells form a uniform population of acinar structures as previously described [15], [32] whereas the three DCIS models form larger and less uniform structures (Fig. 1A).

We performed whole transcriptome sequencing by RNA-Seq for differential transcript expression profiling from two biological replicates of each of the DCIS models (MCF10.DCIS, SUM102 and SUM225) and the MCF10A model of non-tumorigenic mammary epithelial cells. The reads obtained from each sample were grouped to generate clusters and mapped back to the genome as well as the corresponding genes. Using Novoalign software, greater than 80% alignment to the reference genome was observed for all the samples. The number of reads and clusters for each of the RNA-Seq samples is shown in Table S2. Based on a |log_2_ (fold change)| ≥2 and adjusted p-value <0.001, we identified 1,103 genes in MCF10.DCIS, 2,388 genes in SUM102 and 3,036 genes in SUM225 as significantly differentially expressed in comparison to MCF10A. Volcano plots from each individual model are depicted in Figure S1. The plot for the isogenic comparison of MCF10A *vs*. MCF10.DCIS emphasizes that the differentially expressed genes are enriched for those that are common to all three DCIS models (shown in the blue circles), whereas the plots for the SUM models show a broader scatter and more uniquely differentially expressed genes (shown by the green dots).

Hierarchical clustering analysis was carried out to assess the relatedness of the different DCIS models (MCF10.DCIS, SUM102 and SUM225) and non-tumorigenic mammary epithelial cell model (MCF10A). Unsupervised clustering based on the total number of sequence reads shows that, as one would expect, the two biological replicates have a very high correlation coefficient and that there is also a good correlation between the expression profile of the isogenic MCF10A and MCF10.DCIS models (Figure 1B). When clustering is based on differentially expressed transcripts (Figure 1C), the MCF10.DCIS samples are much less correlated with MCF10A samples as compared to when total transcripts are considered. The DCIS models derived from two individual patients (SUM102 and SUM225) are more highly correlated when clustering is based on differentially expressed transcripts (Figure 1C) as opposed to the total number of reads (Figure 1B).

In order to focus our analysis on networks and pathways that most likely underlie breast DCIS, we determined the sub-set of differentially expressed transcripts that were common to all three DCIS models as compared to the MCF10A model of non-tumorigenic mammary epithelial cells (Figure 1D). The results showed that there were over 1,000 differentially expressed transcripts in each of the DCIS models as compared to MCF10A (Figure 1D), with the fewest number of altered transcripts in MCF10.DCIS as compared to MCF10A. Intersection analysis showed that just 25% of the changes from MCF10A to MCF10.DCIS were shared in the independent models provided by SUM102 and SUM225, with 295 significantly differentially expressed transcripts common to all three DCIS models (Table S3). Among these, 63 genes were significantly up-regulated in all three models, 156 genes were down-regulated, and 76 genes showed differential expression but their pattern of up and down regulation was not consistent. The genes identified by RNA-Seq as differentially expressed to the largest extent in all three models of DCIS are depicted with their fold change values in Table 1.

**Table 1:** Genes with the largest fold change in expression identified in the list of those differentially expressed.

Log Ratio up-regulated		Log Ratio down-regulated	
Molecules	Exp Value	Molecules	Exp Value
**AGR2**	**↑** 5.011	**ADH1B**	**↓** −10
**GRHL2**	**↑** 4.435	**NID1**	**↓** −8.728
**APOD**	**↑** 4.239	**DARC**	**↓** −8.382
**CCL20**	**↑** 4.219	**ASPA**	**↓** −8.323
**CHAC1**	**↑** 4.161	**CADM3**	**↓** −8.173
**CLDN4**	**↑** 3.97	**DPT**	**↓** −8.058
**LCN2**	**↑** 3.87	**TLL1**	**↓** −7.994
**LIPH**	**↑** 3.855	**PDPN**	**↓** −7.757
**FUT3**	**↑** 3.804	**TGFBI**	**↓** −7.586
**CREB5**	**↑** 3.714	**RNY5**	**↓** −7.581

The values in the Table refer to log_2_ based fold changes. For any differentially expressed gene, if its log_2_fold change value is infinity (or minus infinity) in any model, it was assigned a value of 10 (or −10). The significantly differentially over-expressed in DCIS in comparison to non-tumorigenic MCF10A include anterior gradient homolog 2 *(AGR2*), grainyhead like (Drosophila) *(GRHL2)*, apolipoprotein D (*APOD*), chemokine (C-C motif) ligand 20 (*CCL20*), cation transport regulator homolog 1 (E. coli) (*CHAC1*), claudin 4 (*CLDN4*), lipocalin 2 (*LCN2*), lipase member H (*LIPH*), fucosyltransferase 3 (*FUT3*), and cAMP responsive element binding protein 5 (*CREB5*). The genes which were found be significantly down-regulated include alcohol dehydrogenase 1B (class I), (*ADH1B*), RNA, Ro-associated Y5 (*RNY5*), transforming growth factor, beta-induced (*TGFBI*), podoplanin (*PDPN*), tolloid-like 1 (*TLL1*), dermatopontin (*DPT*), cell adhesion molecule 3 (*CADM3*), aspartoacylase (*ASPA*), Duffy blood group, chemokine receptor (*DARC*) and nidogen 1 (*NID1*).

### Biological Functions and Pathways Related to Differentially Expressed Transcripts

The DCIS and non-tumorigenic mammary epithelial cell model transcriptomes revealed by RNA-Seq were subject to Ingenuity Pathway Analysis (IPA) to define those pathways that are likely relevant during premalignant progression. This revealed three statistically significant cell signaling and metabolic pathway networks (Table S4). Each network includes the consistently differentially expressed genes as focus molecules along with other related genes from the IPA database. The first network relates to connective tissue disorders, genetic disorders, and dermatological diseases and includes 74 focus molecules or genes with a statistical significance score of 131. The second network is concerned with cellular development, lipid metabolism and molecular transport and includes 43 significantly differentially expressed genes. The third most significant network pertains to lipid metabolism, small molecule biochemistry, organismal injury and abnormalities. This network contains 26 focus molecules from the list of differentially expressed genes. Some of the genes involved in this network are *ABTB2, ADH1B, ALDH5A1, ASPA, CALML3, CHST2, ETNK2, FUT3, GGT6, HIP1, KCNB1, LRRK2, MMP28, PLCH2, RNY5,* and *SEMA5B.* Major canonical pathways and functions associated with the consistently differentially expressed genes are summarized in Table 2, along with molecular and cellular functions and associated diseases and disorders (Table 3). For example, this analysis emphasized the functions of cell-cell signaling and interaction, cellular movement, and cellular organization and assembly and the disease of cancer as significantly associated with the DCIS gene signature. Indeed, many of the significant consistently differentially expressed genes have previously been implicated in cancers other than breast (Table 4).

**Table 2:** Significant canonical pathways associated with differentially expressed genes.

Ingenuity Canonical Pathways	-log(*p*-value)
Atherosclerosis Signaling	3.73E00
Leukocyte Extravasation Signaling	2.79E00
ATM Signaling	1.95E00
Tight Junction Signaling	1.88E00
Fc Epsilon RI Signaling	1.86E00
PTEN Signaling	1.79E00
Glycosphingolipid Biosynthesis	1.79E00
IL-17A Signaling in Gastric Cells	1.68E00
IL-4 Signaling	1.64E00

The list of genes identified as consistently differentially expressed in all models of DCIS were uploaded into Ingenuity Pathway Analysis software. A Fisher’s exact test was used to test the statistical significance with a significance level of 0.05.

**Table 3:** The molecular and cellular functions and diseases and disorders associated with the consistently differentially expressed genes derived from RNA-Seq data analysis.

Molecular and Cellular Functions	*P* -value	No. of molecules
Cell-To-Cell Signaling and Interaction	1.96E-09 - 1.08E-02	35
Cellular Movement	1.97E-07 - 1.08E-02	43
Cellular Assembly and Organization	4.44E-06 - 1.11E-02	22
Cellular Function and Maintenance	4.44E-06 - 8.94E-03	11
Cellular Development	1.04E-05 - 1.11E-02	53
**Diseases and Disorders**	***P*** ** -value**	**No. of molecules**
Cancer	4.23E-09 - 1.07E-02	71
Inflammatory Disease	7.96E-07 - 9.89E-03	60
Connective Tissue Disorders	1.91E-06 - 8.94E-03	42
Genetic Disorder	3.40E-06 - 1.11E-02	100
Dermatological Diseases and Conditions	4.96E-06 - 8.94E-03	31

The values in the second column represent the statistical significance score (*p *value). The number of differentially expressed genes in the dataset are indicated in the third column.

**Table 4:** Significant consistently differentially expressed genes involved in various types of cancers.

Functions Annotation	p-Value	Molecules	No. of Molecules
carcinoma	5.06E-06	ADH1B, AGR2, ALDH5A1, APOD, APOE, BEX2, BGN, C1R, CD70, CDH13, COL1A1, COL7A1, CSF1,FGFR3, GPR56, HIP1, KRT14, KRT7, LCN2, MAPK13, MSLN, MT1E, MYL9, PDPN, POSTN,RAP1GAP, SCD, SERPINF1, TIMP3, TLL1, TNFRSF12A, TP73, TWIST1	33
malignant tumor	1.93E-05	ADH1B, AGR2, ALDH5A1, APOD, APOE, BEX2, BGN, C1R, CD70, CDH13, COL1A1, COL7A1, CSF1,DCN, FGFR3, FOXO1, GPR56, HIP1, IGH@, KRT14, KRT7, LCN2, MAPK13, MSLN, MT1E, MYL9,PDPN, POSTN, RAP1GAP, SCD, SERPINF1, TIMP3, TLL1, TNFRSF12A, TP73, TWIST1	36
digestive organ tumor	2.57E-05	ADH1B, APOE, BGN, COL1A1, CSF1, FGFR3, KRT14, KRT7, LCN2, MAPK13, MSLN, PDPN, POSTN, RAP1GAP, SCD, TIMP3, TP73, TWIST1	18
genital tumor	2.77E-04	APOD, APOE, C1R, CDH13, COL7A1, CSF1, FGFR3, GPR56, HIP1, KRT7, LCN2, MYL9, PDPN, TIMP3,TP73	15
oral cancer	3.40E-04	BGN, COL1A1, KRT14, MAPK13, PDPN, S100P	6
breast cancer	5.23E-04	AGR2, APOE, BEX2, CD70, CDH13, CLDN4, COL1A1, CSF1, ETNK2, FGFR3, FOXO1, HIST2H2BE,KRT14, LCN2, MT1E, PDGFB, PDPN, SERPINF1, TLL1, TP73, TSPAN15	21
head and neck tumor	5.51E-04	ALDH5A1, BGN, FGFR3, KRT14, LAMB1, MAPK13, PDGFB, PDPN, POSTN, RAP1GAP, TP73, TWIST1	12
prostate cancer	5.58E-04	AGR2, ALDH5A1, APOD, C1R, CDH13, COL1A1, COL7A1, CSF1, FGFR3, GPR56, HIP1, KRT7, MYL9,TIMP3, TLL1	15
prostatic carcinoma	7.39E-04	APOD, C1R, CDH13, COL7A1, CSF1, GPR56, HIP1, KRT7, MYL9, TIMP3	10
uterine tumor	7.93E-04	ADH1B, CDH13, CSF1, DPT, DST, FGFR3, HNMT, SPSB1, TIMP3, TP73	10
benign tumor	8.06E-04	ADH1B, DARC, DCN, DPT, DST, FGFR3, HNMT, LCN2, MAPK13, SCD, SEMA5B, SPSB1, TGFBR3, TNFRSF12A, WISP2, ZEB2	16
pancreatic tumor	9.05E-04	BGN, FGFR3, KRT7, LCN2, MAPK13, MSLN, TWIST1	7
colorectal cancer	1.15E-03	C1R, CLDN4, COL2A1, FGFR3, FUT3, HSD17B11, HTRA1, LIPH, MT1E, PDPN, RAP1GAP, SECTM1, SLC2A14, TGFBR3, TIMP3, TJP3, TNIK, TWIST1, WISP2	19
metastasis of lung	2.15E-03	CSF1, LCN2	2

The number of focus genes and the p-values are indicated in the table.

**Figure 2:**
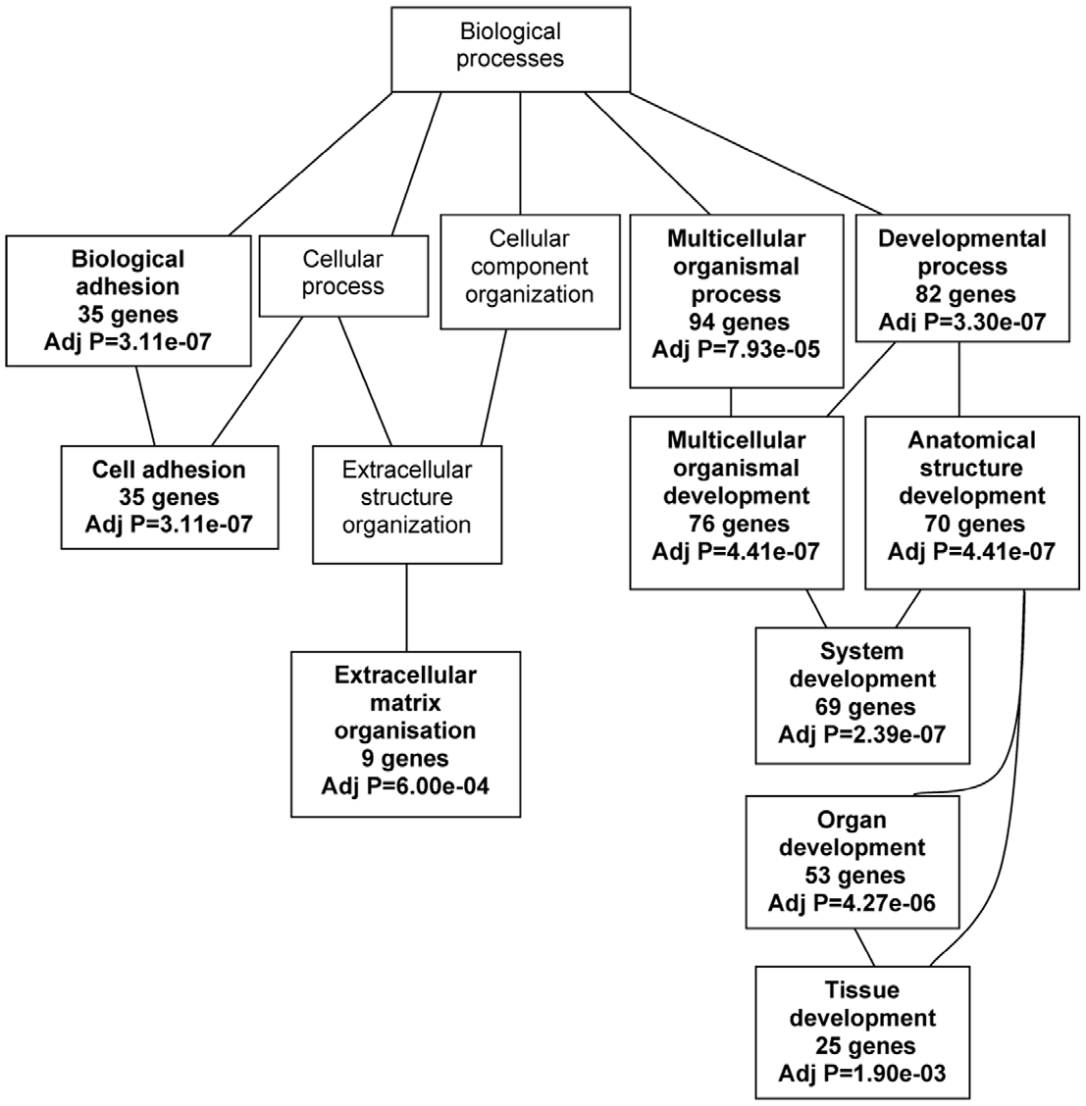
Significant biological processes associated with the differentially expressed genes as identified by Genomatix. The differentially expressed genes in all models of DCIS in comparison to MCF10A were analyzed by WebGestalt2 tool. A Fisher’s exact test was used to determine statistical significance with a significance level of 0.05. The *p*-values and the number of genes associated with a particular function or process are indicated in bold in the respective boxes.

WebGestalt2 [33] and Genomatix GePS were also employed to identify biological processes related to the common significant transcripts from the RNA-Seq data. These two other tools independently mine the relationships and thus yield their own curated data sets. The results showed that a large number of the differentially expressed genes are involved in cell adhesion, cell proliferation, response to chemical and organic stimuli, lipid metabolic processes, organ development and morphogenesis. The corresponding biological processes are summarized in Figure 2 with the number of associated genes and the statistical significance. Their associated molecular functions include protein binding, receptor binding, fibroblast growth factor receptor activity, enzyme regulatory activity, type II transforming growth factor beta (TGFβ) receptor binding, platelet derived growth factor binding and glycosaminoglycan binding.

**Figure 3:**
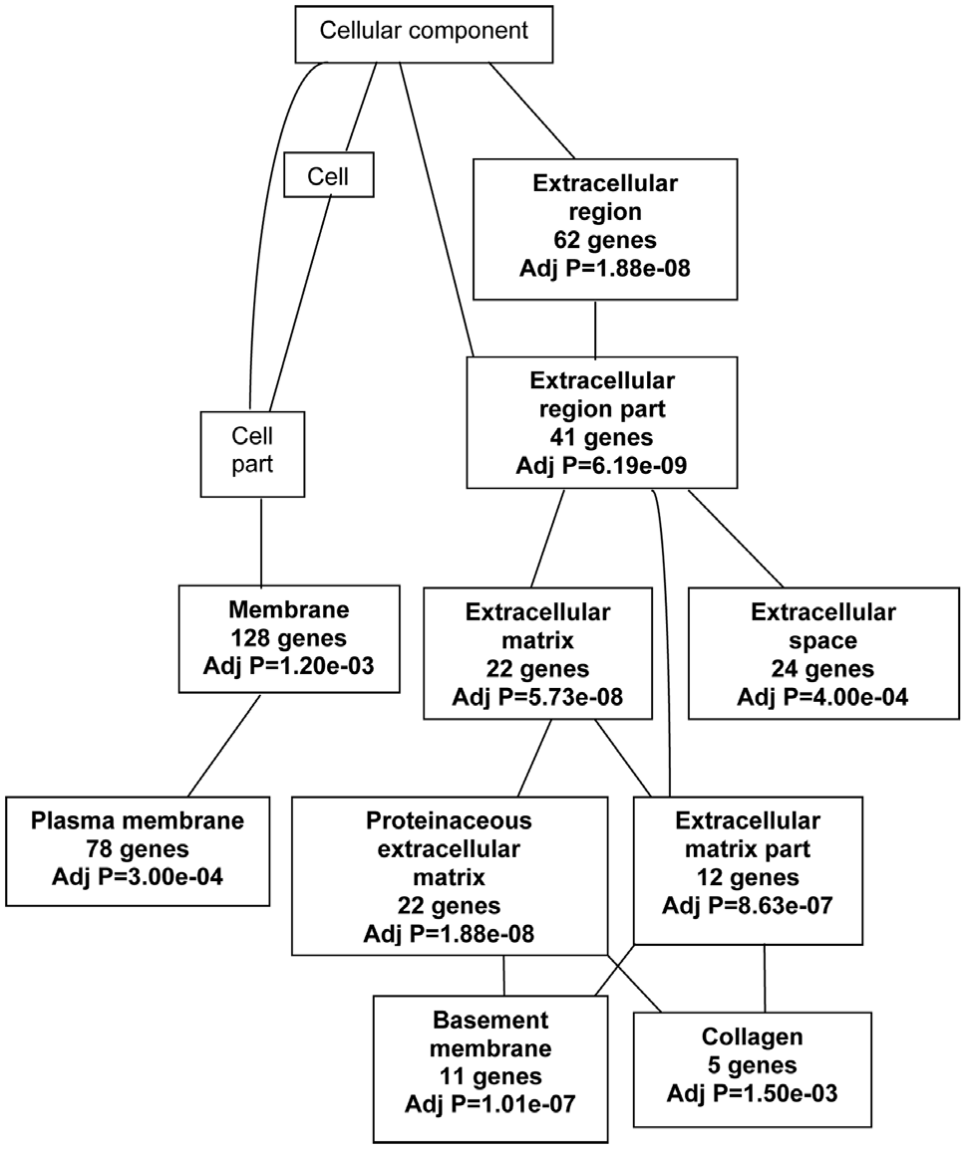
Cellular components related to significantly differentially expressed genes. The differentially expressed genes in all models of DCIS in comparison to MCF10A were analyzed by WebGestalt2 tool. The *p*-values and the number of differentially expressed genes associated with a particular cellular component are indicated in bold in the respective boxes.

Classification of the differentially expressed genes from the DCIS models by cellular component revealed a striking focus of gene expression changes in the plasma membrane and extracellular region (Figure 3). Overlaying GePS also revealed 11 pathways to be most relevant to the differentially expressed genes in DCIS (Table 5). These pathways were dominated by signaling events mediated through integrins, patched homolog1, fibroblast growth factor, TGFβ, hepatocyte growth factor, signal transducer and activator of transcription (STAT) and RhoA.

**Table 5:** Network pathway analysis of differentially expressed genes by Genomatix.

Pathways	*P*-value
Integrin signaling pathway	5.99e-03
Patched homolog 1 (drosophila)	4.54E-04
Fibroblast growth factor	5.41E-04
TGF beta	5.43E-04
Phosphatidylinositol	7.21E-04
Mothers against dpp homolog	1.68E-03
Rhoa ras homolog	2.80E-03
Indian hedgehog v akt murine thymoma viral oncogene homolog 1	3.10E-03
V akt murine thymoma viral oncogene homolog 1	5.55E-03
Hepatocyte growth factor receptor	5.79E-03
Signal transducer and activator of transcription	8.94E-03

The genes identified as differentially expressed in all models of DCIS were analyzed by Genomatix software. A Fisher’s exact test was used to test the statistical significance with a significance level of 0.05.

### qRT-PCR Validation of Key Differentially Expressed Transcripts

**Figure 4:**
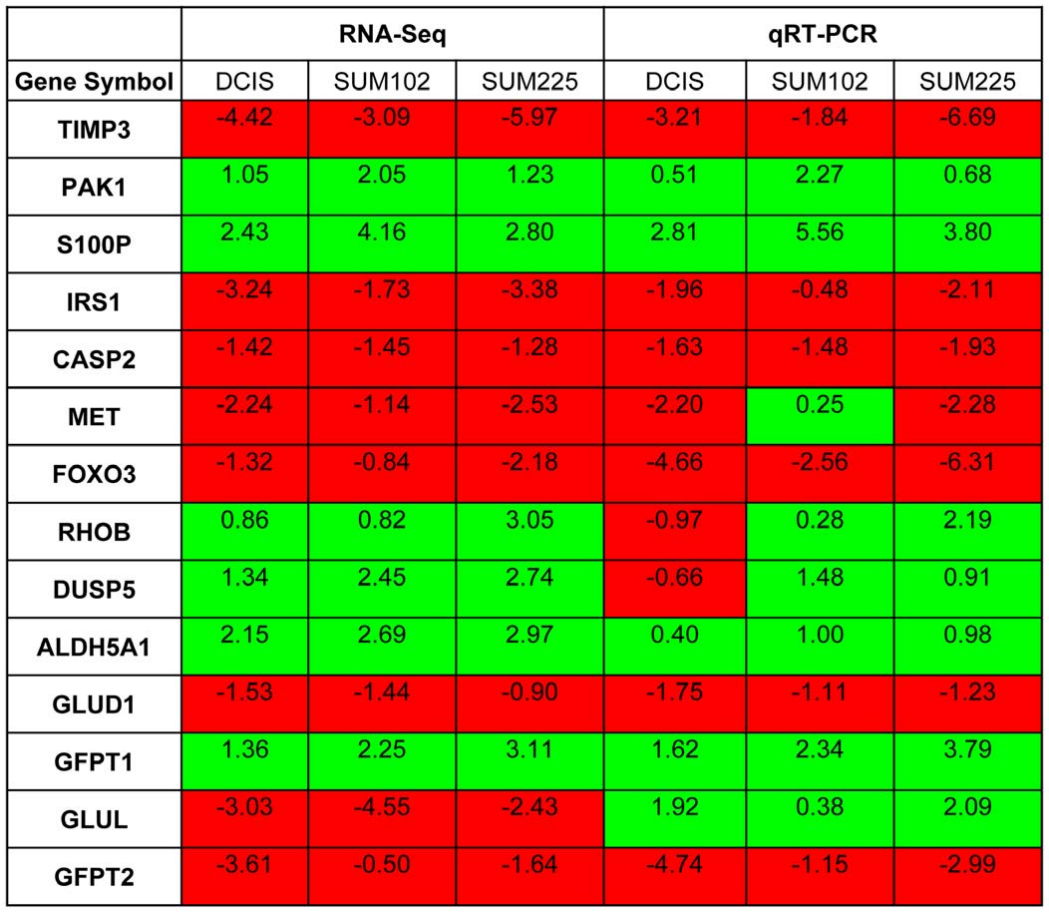
Comparison of the changes in expression of selected genes detected by RNA-Seq and qRT-PCR. The values represent log_2_fold change in MCF10.DCIS, SUM102 and SUM225 compared to expression in MCF10A. Positive values indicate up-regulation (highlighted in green) and negative values indicate down-regulation (highlighted in red).

We employed qRT-PCR to confirm the differential expression of a panel of 14 genes. These genes were selected based on their association with the identified canonical and biological pathways. Expression was normalized to three housekeeping genes (HPRT1, β-actin, and GUSB). The qRT-PCR results were generally in concordance with the sequencing results, with an overall correlation of 0.86. The log_2_fold change values of the selected genes in MCF10.DCIS, SUM102 and SUM225 over MCF10A as detected by RNA-Seq and qRT-PCR are shown in Figure 4. Of the 14 genes selected for qRT-PCR, 10 showed identical directions of change in expression in all three DCIS models in the RNA-Seq analysis. Only one gene, GLUL, was fully discordant, being decreased in all three models by RNA-Seq analysis, yet increased in all three models by qRT-PCR analysis. This observed discrepancy was attributed to non-specific amplification as confirmed by melt curve analysis.

Since MCF10A are immortal, non-tumorigenic mammary epithelial cells that have acquired a number of genetic changes [34], we also assayed the expression of 13 of the above genes in normal primary human mammary epithelial cells (HMEC) by qRT-PCR (Table S5). Six out of the 13 differentially expressed genes that were derived from reference to MCF10A showed some differences in direction of change when the alternative reference to HMEC was used. This discrepancy indicates that these results were affected by variation between individual reference samples. The use of multiple non-tumorigenic references would help to exclude genes that are deregulated in a particular reference model from being identified as significantly differentially expressed in the DCIS models.

### *ALDH5A1* as a Novel Potential Target in Premalignant Progression

ALDH5A1 was selected as an initial target for detailed investigation for several reasons. First, there was a strong gain of expression in all three models of DCIS that was revealed by RNA-Seq and confirmed by qRT-PCR (Figure 4 and Table S5). Second, ALDH5A1 represented a novel transcript that had not previously been associated with breast cancer. Third, there is established pharmacology for inhibition of ALDH5A1 through two FDA-approved drugs (disulfiram [DSF] [35] and valproic acid [VPA] [36]). Both are known to be safe for chronic use in patients with other disorders but have not previously been considered in the context of DCIS.

**Figure 5:**
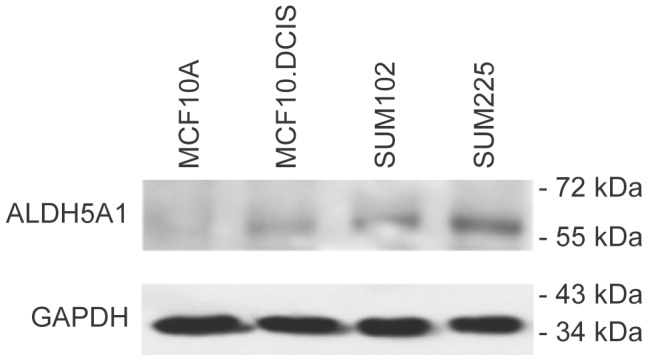
ALDH5A1 is over-expressed in DCIS models. Whole cell lysates from 3D rBM overlay cultures of MCF10A, MCF10.DCIS, SUM102 and SUM225 were probed for ALDH5A1 (upper panel) and for GAPDH (lower panel) as a loading control. Densitometry indicated that ALDH5A1 levels in the lysates of MCF10.DCIS, SUM102, and SUM225 were 2.7, 3.1, and 7.3-fold over that in MCF10A in this representative experiment. In three independent analyses of MCF10.DCIS 3D rBM cultures, the mean increase in ALDH5A1 over control was 3.8-fold.

Changes in mRNA level are not always reflective of changes in protein expression, so we determined the level of ALDH5A1 protein in MCF10.DCIS, SUM102, SUM225 and MCF10A models by immunoblotting (Figure 5). We observed increased expression of ALDH5A1 in all three models of DCIS in comparison to the MCF10A model.

**Figure 6:**
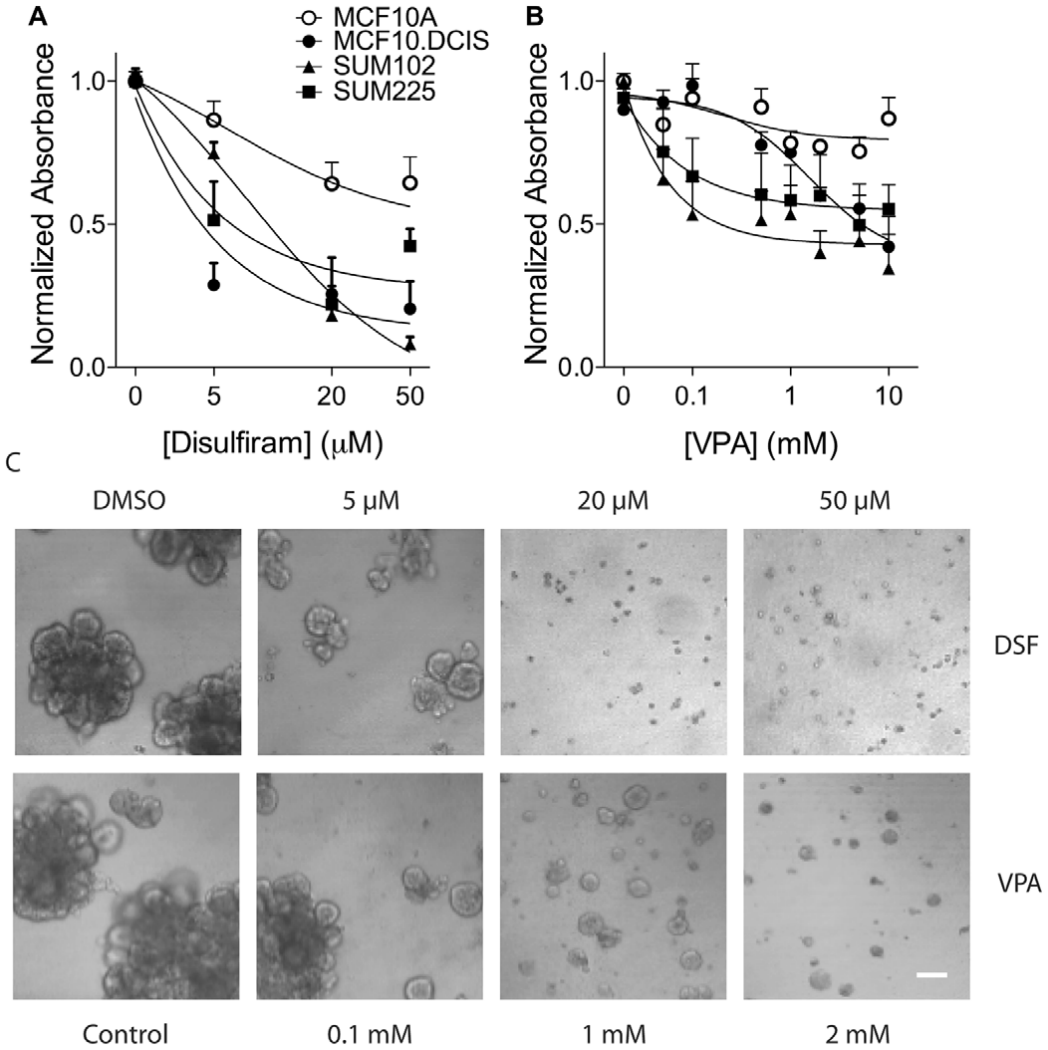
ALDH5A1 as a target in DCIS models. (*A, B*) Effect of DSF and VPA on proliferation of MCF10A (*open circles*), MCF10.DCIS (*closed circles*), SUM102 (triangles) and SUM225 (*squares*) 3D rBM cultures. Cells were incubated for 72 hours with the indicated concentrations of drugs or with DMSO as a vehicle control and subjected to an MTT assay. Sigmoidal dose-response curves were plotted using nonlinear regression analysis. Data represent the mean ± SEM of three independent experiments (each performed in triplicate). (*C*) Effect of DSF and VPA on 3D rBM cultures of SUM102-mRFP cells. Cells were incubated for 8 days with the indicated concentrations of drugs or with DMSO as a vehicle control and DIC images are shown. Size bar, 90 µm.

Disulfiram (DSF) is a broad spectrum and irreversible inhibitor of many isozymes in the aldehyde dehydrogenase family [35]. Treatment with DSF significantly inhibited net proliferation of 3D rBM overlay cultures of MCF10.DCIS, SUM102 and SUM225, but had only a modest effect on 3D rBM overlay cultures of MCF10A (Figure 6A). For example, a concentration of 20 µM DSF significantly (*p*<0.001) reduced the net proliferation of MCF10.DCIS, SUM102 and SUM225 cultures. Whether this observed decrease in cell number upon treatment with DSF was due to decreased proliferation or increased cell death was not explored.

In contrast to DSF, valproic acid (VPA) is a selective blocker of the ALDH5A1 isoform [36] that is increased in the three DCIS models. VPA is also an inhibitor of the histone deacetylases [37], [38], [39]. Treatment with VPA had a minimal effect on the net proliferation of MCF10A 3D rBM overlay cultures at any of the doses tested, but inhibited net proliferation of MCF10.DCIS, SUM102, and SUM 225 3D rBM overlay cultures in a dose-dependent manner (p<0.05) (Figure 6B). The reduction in cell numbers by VPA might result from decreased proliferation or increased cell death or a combination of both.

As an independent assay for effects on cell growth, we developed variants of MCF10.DCIS and SUM102 cells that constitutively expresses monomeric red fluorescent protein (mRFP) and treated 3D rBM overlay cultures of these cells. The results show the dysplastic growth of the control and vehicle-treated MCF10.DCIS-mRFP and SUM102-mRFP cells over a period of eight days. This growth is essentially terminated by inclusion of DSF at ≥20 µM (Figure 6C, Figure S2). We also tested VPA against the SUM102-mRFP cells and found that ≥0.1 mM was sufficient to reduce their net proliferation (Figure 6C). To test whether these growth inhibitory effects of DSF and VPA were reversible, we re-plated the cells in fresh media without drug and assayed their ability to re-grow over 10 days (Figure S3). The SUM102-mRFP cells that had been treated with 20 or 50 µM DSF showed no ability to re-grow, whereas those that had been treated with 5 µM DSF showed minimal re-growth as compared to control, vehicle-treated cells. Treatment with as little as 0.1 mM VPA was sufficient to almost completely block re-growth.

## Discussion

Comprehensive gene expression profiling has been used extensively for molecular classification of invasive breast tumors, but relatively fewer studies have been performed in DCIS [20], [21], [22], [23], [24], [25], [26], [27], [28], [40], [41]. DCIS is commonly classified as premalignant and so may have been viewed as a lower priority for study despite its increased diagnosis in the population, and even though some of these lesions may be predetermined to eventual malignancy [42]. We used novel and tractable models of DCIS in an *in vitro* 3D rBM overlay culture system originally developed to study morphogenesis and oncogenesis of MCF10A cells [43]. The 3D rBM overlay cultures are a better mimic of the *in vivo* environment than cells grown on plastic dishes [44] and also provide a source for high quality RNA with avoidance of the contribution of stromal cell RNA. Both of those latter advantages are hard to achieve in the isolation of RNA from microscopic clinical DCIS specimens or from formalin-fixed paraffin-embedded tissues. Here, we used high-throughput next generation sequencing for global transcriptome profiling of these novel DCIS models. To the best of our knowledge, this is the first report of next generation sequencing in 3D rBM overlay culture models of DCIS of the breast.

Several molecular markers that predict the risk of recurrence in patients with DCIS have previously been defined. Estrogen receptor (ER) expression is inversely related to the grade of DCIS lesions [8] and targeting DCIS that express ER with tamoxifen significantly reduces risk of subsequent breast cancer by 40%–50% [45]. Progesterone receptor (PR) expression also has an inverse relationship to nuclear grade and its presence is associated with expression of ER and lack of comedo-necrosis in DCIS [46], [47]. All of the models of DCIS (MCF10.DCIS, SUM102, SUM225) used in the present study lack ER and PR expression and represent aggressive disease for which conventional anti-estrogen therapies (including tamoxifen and raloxifene) would not be used. Her-2/neu amplification plays an important role in initiation rather than in progression of ductal carcinoma [48] and its overexpression predicts local recurrence [49]. SUM225 cells have amplification of Her2/neu. A direct positive relationship has been observed for the expression of ER, PR, and Bcl-2 [50]. Ringberg *et al.* [51] suggest that a molecular signature with lack of ER and PR, Her2 over-expression, accumulation of p53, and high ki67 expression is a strong predictor of local recurrence rate in DCIS. In a retrospective study of DCIS cases, DCIS lesions that were positive for p16, COX-2, and Ki67 expression were significantly associated with risk of subsequent invasive cancer whereas DCIS lesions that either lacked ER but were positive for ERBB2 and Ki67 or that lacked COX2 and were positive for p16 and Ki67 were associated with recurrence of DCIS [52].

### Differentially Expressed Genes in Models of DCIS

Sequencing revealed many novel differentially expressed transcripts in DCIS. The hierarchical clustering of samples indicates the robustness of the data and the reproducibility of the biological replicates. The observation that MCF10.DCIS has more genes in common with MCF10A than with either SUM102 or SUM225 is consistent with the fact that MCF10.DCIS and MCF10A are isogenic. The implication of the majority of the differentially expressed genes in the processes of cell-cell adhesion, cell proliferation and movement signify the importance of deregulation of these processes very early in the course of premalignant progression. Interestingly, several of the ten most down regulated molecules in the list of significantly differentially expressed genes pertain to the functions of cell adhesion and include CADM3 (cell adhesion molecule 3), DPT (dermatopontin), NID1 (nidogen1) and TGFBI (transforming growth factor beta induced). The latter is consistent with the previous observation that the level of TGFBI decreases in progression from benign breast tissues to DCIS and IDC [53]. TGFBI activates adhesion-associated signaling and decreases the motility in breast cancer cells both *in vitro* and *in vivo*. It also reduces the activation of matrix metalloproteinases (MMPs) 2 and 9, which are responsible for the degradation of extracellular matrix [54].

In contrast to decreased TGFBI that has previously been associated with DCIS, decreased CADM3, DPT, and NID1 have not previously been linked to breast cancer. CADM3 also known as nectin like protein 1 (Necl1) is a cell-cell adhesion molecule and has been reported to suppress tumorigenicity in colon cancer cells [55]. Loss of its expression has been detected in various gliomas [56]. DPT is involved in cell adhesion and promotes ECM assembly. Downregulation of DPT has been previously observed in oral squamous cell carcinoma and is associated with lymph node metastasis [57]. Decreased expression of DPT in hepatocellular carcinoma has also been reported [58]. We also observed greater than tenfold down regulation in expression levels of *NID1* (Nidogen-1). Nidogens provide structural stability to basement membrane by connecting laminin and collagen IV networks. They interact with various integrins and play an important role in cell adhesion. Loss of nidogen expression weakens the basement membrane and favors invasion. A study by Ulazzi and co-workers reported that loss of NID1 expression observed in colon and gastric tumors is due to aberrant methylation of the NID1 promoter [59], whereas a genome-wide association study by Nan et al. [60] identified NID1 as a susceptibility locus for melanoma.

Some of the significantly over-expressed genes found in DCIS included AGR2 (anterior gradient 2), CLDN4 (claudin 4) and LCN2 (lipocalin 2). The presence of AGR2 in primary breast tumors is correlated with poor survival [61], and elevated expression of AGR2 is related to treatment failure with tamoxifen [62]. High levels of CLDN4 have been reported in basal-like breast cancers [63]. In gastric carcinoma, high levels of CLDN4 have been found to be significantly associated with MMP-9 expression, which in turn can degrade type IV collagen of ECM and facilitate cancer cell invasion [64]. In comparison, increased LCN2 promotes breast cancer progression and metastasis by facilitating epithelial-to-mesenchymal transition [65].

Several findings from our sequencing study are in agreement with results from other gene expression profiling studies in DCIS. Down regulation of DST and HTRA1 has been associated with progression to invasive breast cancer [66], [67] and was found in our data-set. Similarly, upregulation of GJB2 expression, which is involved in local invasion of breast ductal carcinoma [68], was also found. We observed differential gene expression of several collagens (1A1, 2A1, L4A6, 7A1,8A1 and 17A1) in DCIS models, but not the related family members (e.g.,11A1 and 5A2) that have previously been reported to be involved in progression of DCIS to IDC [69]. Similar to the findings of comparative microarray analyses of MCF10A and MCF10.DCIS trancriptomes [70], we found up regulation of ABTB2, CX3CL1, DHRS9, GRHL2, HNMT, KRT6B, KRT7, LCN2, MYEOV, PLEKHF1, SEMA4A, and TNFRSF12A; down regulation of APOE, C1R, COL4A6, D4S234E, KRT14, and PCDH7; and differential expression of several members of various gene families like ALDH, CAPN, CCDC, CHST, ITGB, SLC and TMEM.

### ALDH5A1 in Premalignant Progression

The sequencing results presented in this study showed that several differentially expressed genes in DCIS are members of the glutamate metabolic pathway. These include aldehyde dehydrogenase 5 family member 1 (*ALDH5A1*), glutamine–fructose-6-phosphate transaminase 2 (*GFPT2*), glutamate dehydrogenase 1 (*GLUD1*), and glutamate ammonia ligase (*GLUL*). Although a link between glutamate metabolism and premalignant breast disease would be a novel connection in the context of DCIS, metabolomic profiling of gastric cancer metastases also identified associations with glutamate metabolism [71].

From among the group of differentially expressed gene products in the DCIS models that are involved in glutamate metabolism, we chose to focus on ALDH5A1 due to its novelty in the context of breast cancer and status as an established druggable target. The gene product of *ALDH5A1*, also known as succinic semi-aldehyde dehydrogenase (SSDH), is involved in the catabolism of neurotransmitter 4-aminobutyric acid (GABA) and is highly expressed in brain. ALDH5A1 belongs to the superfamily of aldehyde dehydrogenases (ALDHs) that oxidize aldehydes to the corresponding carboxylic acids. Very little is known about *ALDH5A1* with regard to cancer, beyond an observation in renal cell carcinoma cells that it is regulated by hepatocyte nuclear factor 4alpha [72]. In contrast, elevated activity of the ALDHs 1A2, 1A3, 1A7, 3A1, 4A1, 5A1, 6 and 9A1 has been reported in stem cells [73]. Increased activity of ALDH1A1 has been observed in the stem cell populations of multiple myeloma, acute myeloid leukemia and malignant mammary cells [74], [75]. Downregulation of ALDH1A1 and ALDH3A1 has been shown to reduce cell growth and motility in lung cancer cells [76]. ALDH3A2 is one of the 35-gene signature that is reported to discriminate between well- and poorly differentiated DCIS [28].

The RNA-Seq results showed significant increases in ALDH5A1 expression in the DCIS models that was validated by both qRT-PCR and immunoblotting. Meta-analysis of normalized gene expression profiles in the GeneSapiens “body-wide” microarray database reveals that the median level of ALDH5A1 expression is significantly higher in certain cancers (particularly glioma and some leukemias and lymphomas). In the group of breast and reproductive cancers, the median expression of ALDH5A1 remains low. Notably, there are outliers of breast cancers classified as ductal and classified as not-otherwise-specified (which would include DCIS and IDC, respectively) in which ALDH5A1 is increased in expression (Figure S4).

Disulfiram (DSF) is an irreversible inhibitor of aldehyde dehydrogenase enzymes and has notably been shown to block ALDH5A1 in brain slices [35]. DSF has been used clinically for several decades as a deterrent to alcohol consumption and has recently emerged as a potential cancer drug. Its antitumor activity has been previously reported in both *in vitro* and in xenograft studies of breast cancer cell lines [77], [78], but the possibility that this activity could be due to inhibition of the ALDH5A1 isoform was not previously considered. We observed that DSF at low micromolar concentrations was effective in inhibiting net proliferation of all three DCIS models, while having negligible effect on the MCF10A model of non-tumorigenic breast epithelia. Notably, 20 µM DSF had a significant effect on all the DCIS models in 3D rBM culture. This concentration has previously been shown to have no effect on MCF10.DCIS cells grown in conventional 2D cell culture unless supplemented with 20 µM CuCl_2_ to allow inhibition of the proteasome [77]. In the present study, the copper concentration in the medium used for 3D culture was approximately 1 nM. Hence, the observed effect on proliferation is unlikely to be due to inhibition of proteasomal activity. The present data do not, however, distinguish whether the reduction in cell number is due to reduced proliferation, increased cell death or a combination of both effects.

VPA is an FDA-approved drug with a long established history of safe use as an anti-epileptic. It has recently been adapted to treat refractory migraines and psychiatric disorders [79]. VPA inhibits ALDH5A1 with a K_i_ of ∼ 0.5 mM [80], and this activity is important to its anti-seizure activity [79]. More recently VPA has also been found to inhibit histone deacetylases with similar potency [37], [38], [39]. Notably, the serum concentration of VPA in patients under standard chronic therapy is 0.35–0.7 mM [39]. We have observed in our study that VPA, in a concentration dependent manner, inhibits net proliferation in all three models of DCIS while having a minimal effect on the non-tumorigenic breast epithelial cells. As with the inhibition of net proliferation by DSF, the effects of VPA may include reduction in cell proliferation, increase in cell death or both. The concentrations of VPA that effectively reduce DCIS cell numbers are within the therapeutic range for VPA therapy in humans [39]. The discovery of VPA inhibition of histone deacetylases has provided a rationale for studies to test whether it has anti-cancer effects, including in the context of breast cancer [e.g., ClinicalTrials.gov: NCT01010854]. The results from our study suggest that inhibition of ALDH5A1 may contribute to its anti-tumorigenic activity.

In the present study, we have used next generation sequencing to identify the transcriptional fingerprint of three DCIS models at the whole genome level. The data presented reveal significant differentially expressed transcripts, pathways and networks in DCIS. ALDH5A1, an enzyme of glutamate metabolism, has not previously been linked to DCIS. Two drugs, DSF and VPA, that target ALDH5A1 significantly reduced net proliferation in 3D DCIS models. As these drugs are already approved for non-cancer indications, the results presented above suggest that additional *in vivo* studies are warranted to evaluate the potential repurposing of DSF and VPA to treat DCIS.

## Supporting Information

Figure S1 **Volcano plots depicting differentially expressed (DE) genes in the various DCIS models compared with MCF10A.** For each plot the X-axes represent fold change (log_2_) and the Y-axes denote adjusted p-values(-log_10_). Thresholds of |log_2_(fold change)| ≥2 and -log_10_(adjusted p-values) >3 (equal to adjusted p-value <0.001) were applied to identify DE genes. Each dot (or circle) represents a single gene. The black dots indicate genes that were not DE; the blue circles highlight the genes that are DE in all three models compared to MCF10A; the green circles indicate the genes that are DE in that specific pairwise comparison but that are not common to all three models; the red dot corresponds to ALDH5A1. To include all data points, transcripts exhibiting a log_2_(fold change) value of infinity (or minus infinity), is assigned a value of 15 (or -15) and -log_10_(adjusted p-values) of infinity is assigned a value of 300. *Left*, MCF10.DCIS vs. MCF10A; *Middle*, SUM102 vs. MCF10A; *Right*, SUM225 vs. MCF10A.
(DOC)

Figure S2 **Treatment of MCF10.DCIS cells that express mRFP with DSF.** Differential interference contrast images of MCF10.DCIS-mRFP cells cultured in 3D for 8 days in the absence or presence of the indicated concentrations of DSF or DMSO as a solvent control. The images were collected at 10× magnification.
(DOC)

Figure S3 **Quantification of re-growth of SUM102-mRFP cells after drug exposure.** Three-dimensional rBM overlay cultures of SUM102-mRFP cells were treated for 8 days with the indicated concentrations of VPA (*upper panel*) or DSF (*lower panel*) or vehicle control. The cells were then harvested from the matrix and re-plated in fresh media without inhibitors for growth in 2D over 10 days. Results shown are mean ± SEM from three independent experiments.
(DOC)

Figure S4 **Meta-analysis of normalized gene expression profiles in the GeneSapiens microarray database.** The highlighted designations of “Breast, ductal cancer” and “Breast carcinoma, NOS” include DCIS and IDC. The boxes represent the quartile distribution (25–75%) range and the red horizontal lines show the median. The plots also show 95% black whiskers and the individual outlier samples as small circles.
(DOC)

Table S1 **Primer pair sequences of genes quantified for expression levels by real time PCR.**
(DOC)

Table S2 **Generation of clusters from the reads obtained by deep sequencing of different samples.** Biological duplicates of MCF10A, MCF10.DCIS, SUM102 and SUM225 samples were run in the Solexa flowcell. No. of reads indicates the total number of short reads that uniquely aligned to reference genome. The reads from each sample were grouped into clusters using two parameters: 1. window size 100 bp; 2. number of reads per cluster ≥9. No. of clusters indicates those generated from the reads based on Poisson distribution. The reads that did not group in any cluster were considered as background and discarded. Clusters/percentage indicates reads in clusters compared with the total number of reads.
(DOC)

Table S3 **Significantly differentially expressed genes common to all three DCIS models (MCF10.DCIS, SUM102 and SUM225) in comparison to MCF10A.** The values in each column represent the log_2_ fold change in a DCIS model over MCF10A with adjusted *p*-value. “Inf” indicates that no reads mapped to that DCIS model.
(DOC)

Table S4 **Signaling pathway networks involving consistently differentially expressed genes.** Ingenuity Pathway Analysis (IPA) of differentially expressed genes revealed several statistically significant pathway networks. Each network contains focus molecules that are obtained from RNA-Seq analysis and are indicated in bold. The other genes in the network are derived from IPA database. The statistical significance score and top functions associated with the molecules in network are also indicated in the table.
(DOC)

Table S5 **Expression levels of selected genes in three models of DCIS in comparison to normal human mammary epithelial cells (HMEC).** The expression of all genes by qRT-PCR was normalized to that of β-glucuronidase (GUSB) as a housekeeping gene. The values in the table are Log_2_ (Fold Change), positive values indicate up-regulation in the DCIS model (highlighted in green); negative values indicate down-regulation (highlighted in red).
(DOC)
