# Supplementary figures and images for: RNA-Seq of Human Breast Ductal Carcinoma In Situ Models Reveals Aldehyde Dehydrogenase Isoform 5A1 as a Novel Potential Target
Source: PLoS One. 2012 Dec 6;7(12):e50249. doi: 10.1371/journal.pone.0050249 (PMC3516505; doi:10.1371/journal.pone.0050249)

**Figure S1**

**
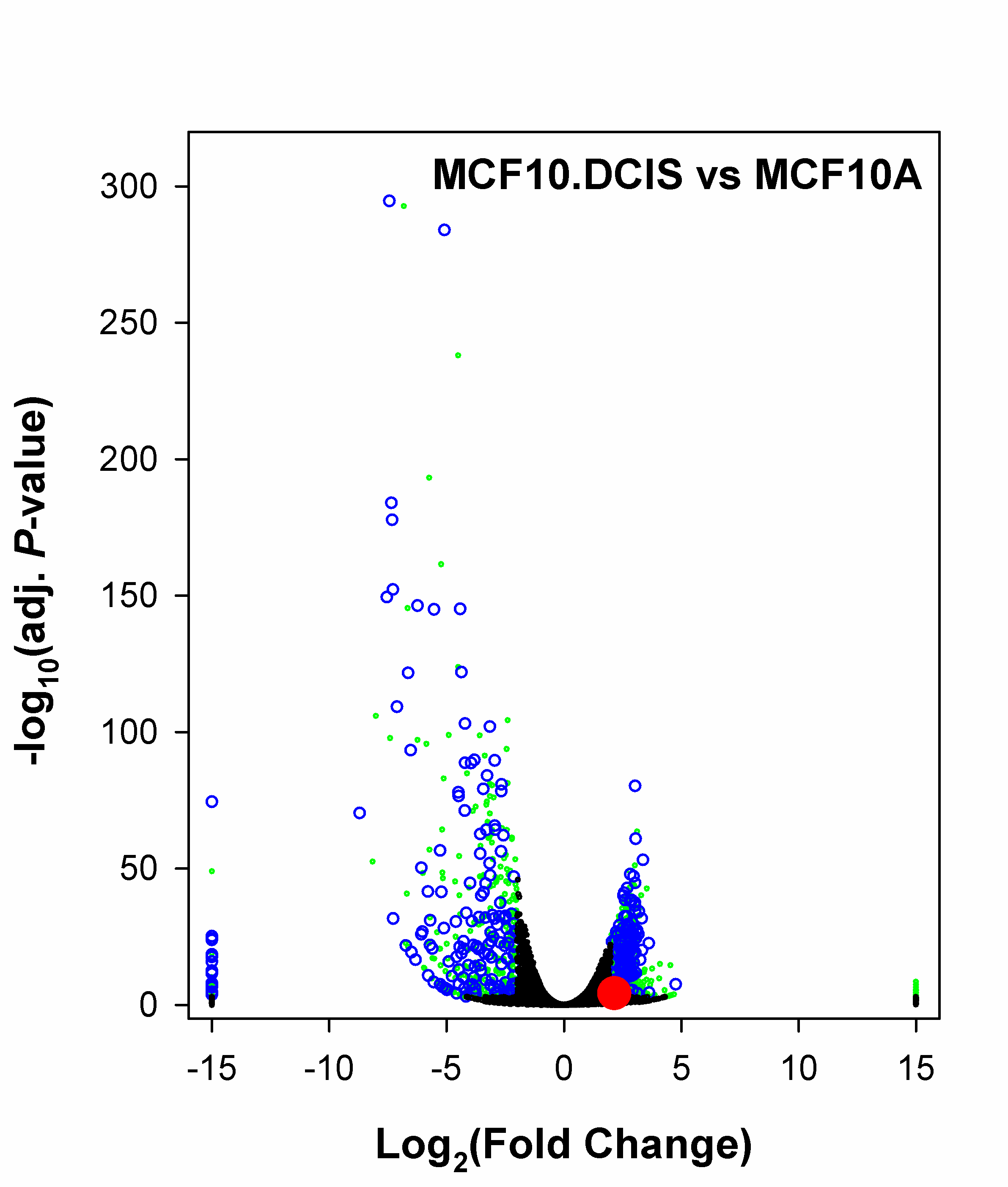

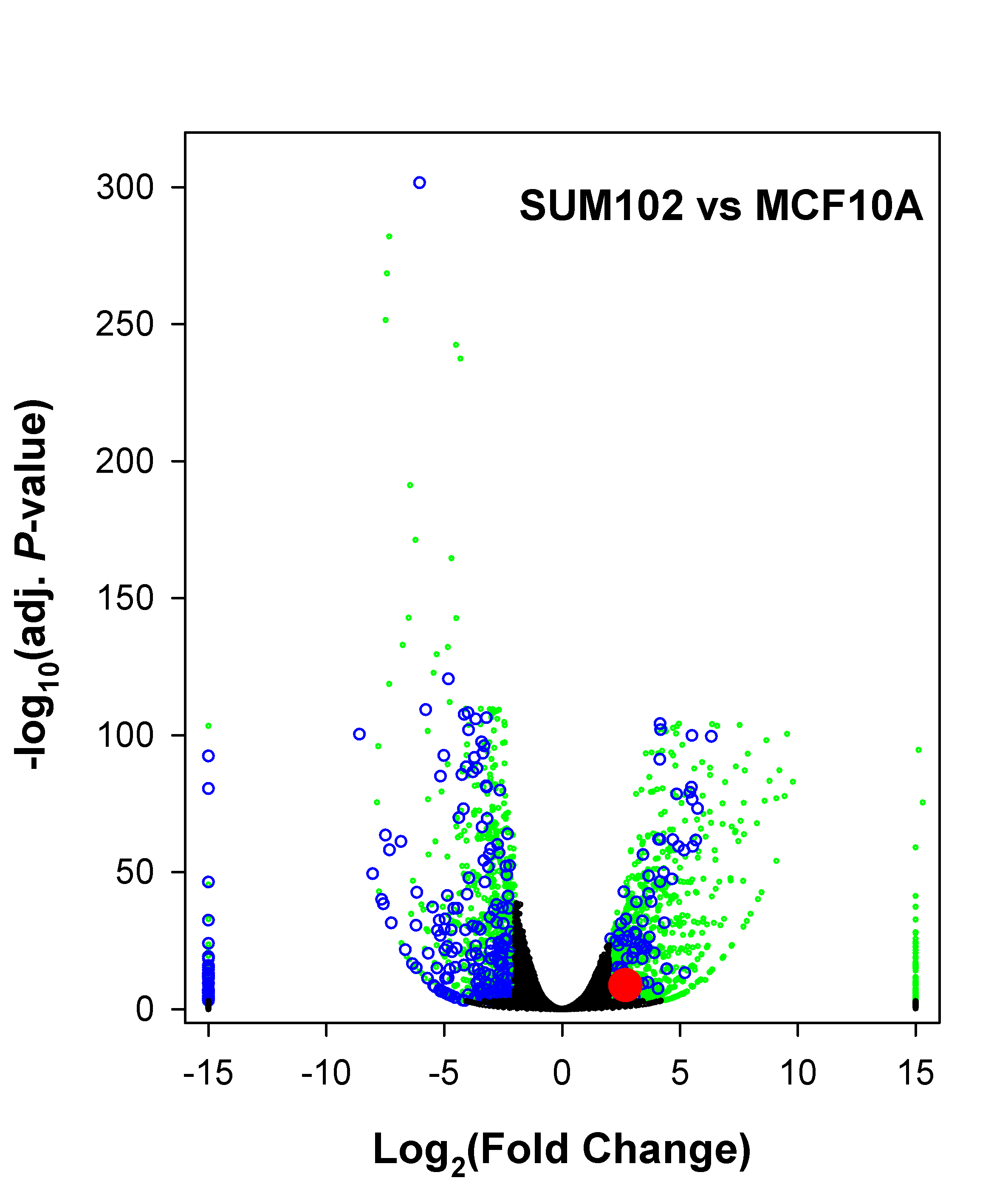

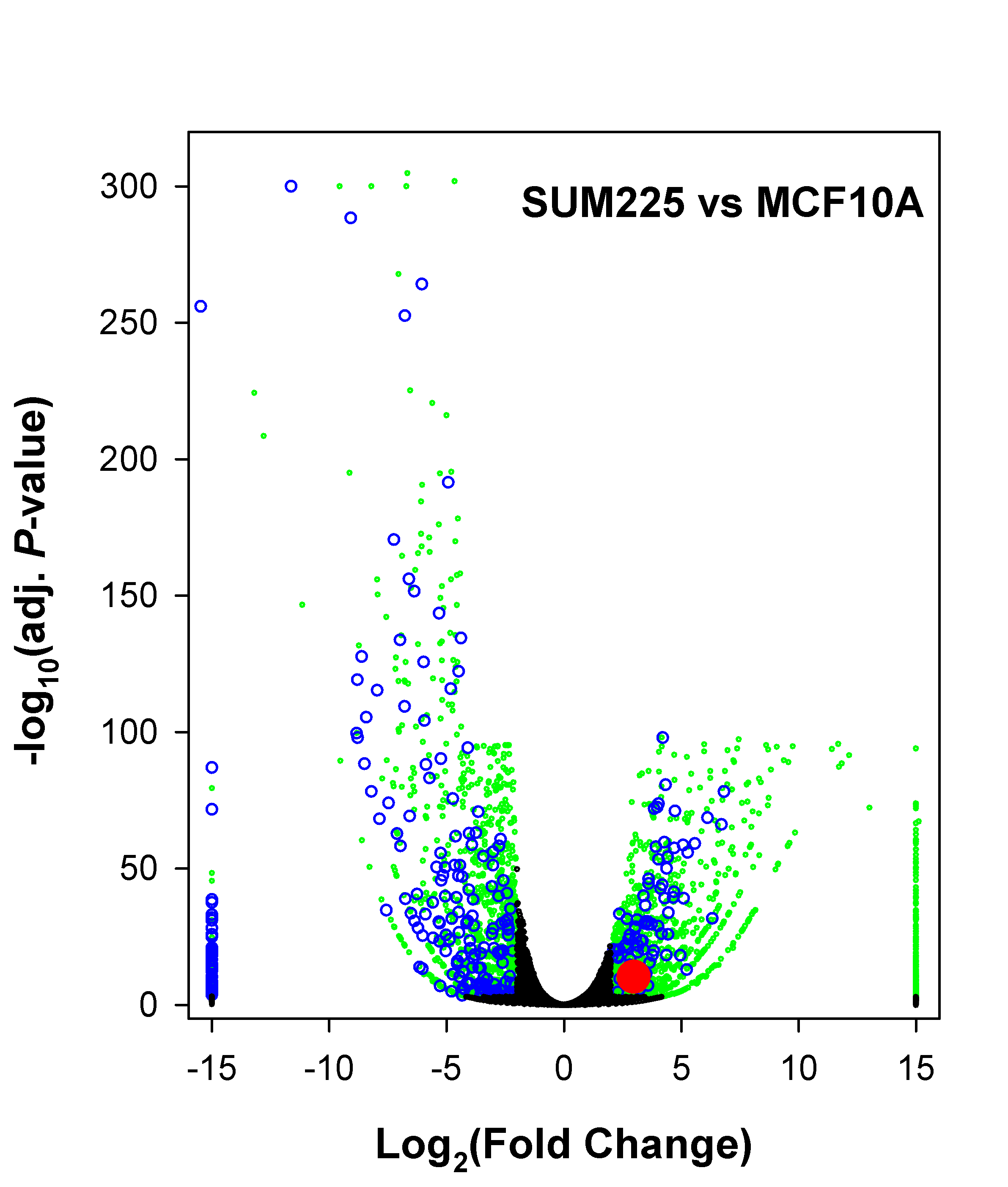
**

Supplement: Figure S1 — Volcano plots depicting differentially expressed (DE) genes in the various DCIS models compared with MCF10A. For each plot the X-axes represent fold change (log2) and the Y-axes denote adjusted p-values(-log10). Thresholds of |log2(fold change)| ≥2 and -log10(adjusted p-values) >3 (equal to adjusted p-value <0.001) were applied to identify DE genes. Each dot (or circle) represents a single gene. The black dots indicate genes that were not DE; the blue circles highlight the genes that are DE in all three models compared to MCF10A; the green circles indicate the genes that are DE in that specific pairwise comparison but that are not common to all three models; the red dot corresponds to ALDH5A1. To include all data points, transcripts exhibiting a log2(fold change) value of infinity (or minus infinity), is assigned a value of 15 (or -15) and -log10(adjusted p-values) of infinity is assigned a value of 300. Left, MCF10.DCIS vs. MCF10A; Middle, SUM102 vs. MCF10A; Right, SUM225 vs. MCF10A. (DOC) [file pone.0050249.s001.doc]

**Figure S2**

**
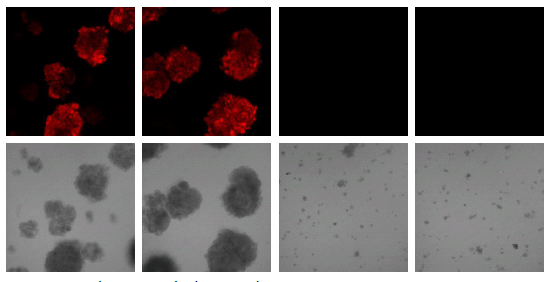
**

50 μM

20 μM

DMSO

Control

Supplement: Figure S2 — Treatment of MCF10.DCIS cells that express mRFP with DSF. Differential interference contrast images of MCF10.DCIS-mRFP cells cultured in 3D for 8 days in the absence or presence of the indicated concentrations of DSF or DMSO as a solvent control. The images were collected at 10× magnification. (DOC) [file pone.0050249.s002.doc]

**Figure S4**

**
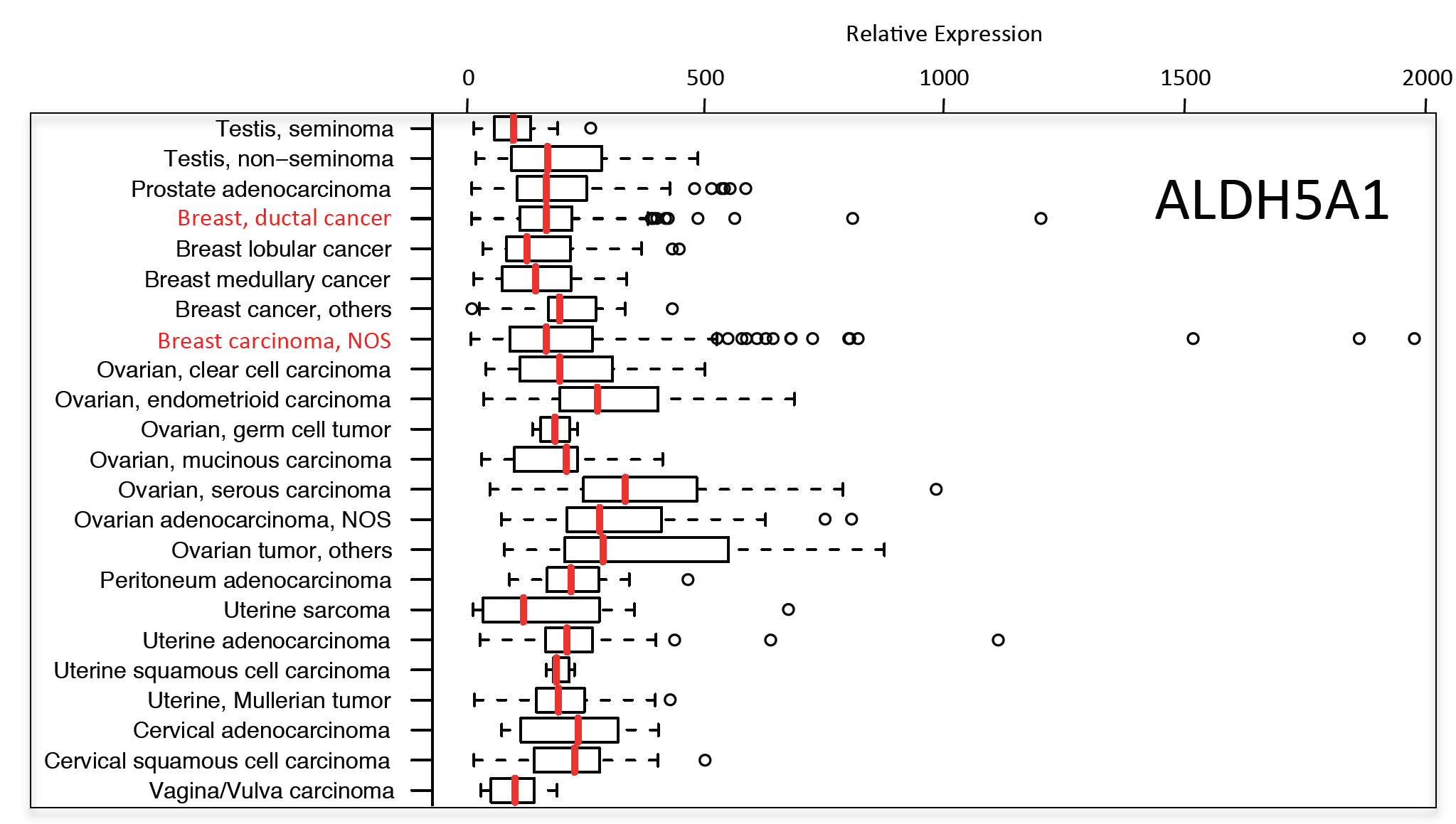
**

Supplement: Figure S4 — Meta-analysis of normalized gene expression profiles in the GeneSapiens microarray database. The highlighted designations of “Breast, ductal cancer” and “Breast carcinoma, NOS” include DCIS and IDC. The boxes represent the quartile distribution (25–75%) range and the red horizontal lines show the median. The plots also show 95% black whiskers and the individual outlier samples as small circles. (DOC) [file pone.0050249.s004.doc]
