# Supplementary material for: RNA-Seq of Human Breast Ductal Carcinoma In Situ Models Reveals Aldehyde Dehydrogenase Isoform 5A1 as a Novel Potential Target
Source: PLoS One. 2012 Dec 6;7(12):e50249. doi: 10.1371/journal.pone.0050249 (PMC3516505; doi:10.1371/journal.pone.0050249)
Supplement: Table S1 — Primer pair sequences of genes quantified for expression levels by real time PCR. (DOC) [file pone.0050249.s005.doc]

**Table S1**

| **Gene of interest** | **Primer Sequence** |
| --- | --- |
| **TIMP-3** | Forward: ACGCTGGTCTACACCATCAAGCAGA  Reverse: GACGCGACCTGTCAGCAGGTACTGG |
| **GFPT-1** | Forward: GTTGGCACAAGGCGAGGTAGCC  Reverse: AAGGCAGGTTGTGCTGTCCACAC |
| **S100P** | Forward: CGATATTCGGGCAGCGAGGGC  Reverse: CTTTTCCACTCTGCAGGAAGCCTG |
| **RHOB** | Forward: CCGGGAGAGAGCTAGGCCGAGT  Reverse: GGATCGGCGGCTTTGTGCGTA |
| **FOXO3** | Forward: TTCGCTGGCCGCACGTCTTCAG  Reverse: GGAGAGTTGGTTATCCCGGGCCG |
| **MET** | Forward: CTTTGCCAGTGGTGGGAGCACA  Reverse: AGCGATGTTGACATGCCACTGTAA |
| **IRS1** | Forward: GGAGTGCACCCCTGAACCGC  Reverse: GGTCTTCATTCTGCTGTGATGTCCA |
| **ALDH5A1** | Forward: GCATAGCCACACCCATTCATT  Reverse: CCAACTATTCAACTCTGCCAAGAA |
| **GLUL** | Forward: CTCGCTCTCGCGGCCTAGCTTT  Reverse: CCTGAGGCAGGGACATGTACACC |
| **GLUD1** | Forward: AGCTTTGGCTTCTCTGATGACAT  Reverse: ACCCCCAAACGGCACAT |
| **CASP2** | Forward: CCCACCGTTGAGCTGTGACTACGA  Reverse: **GGCTTCACCTGAAGGCAGACAGG** |
| **DUSP5** | Forward: ATGACCAGGGTGGCCCAGTTGAA  Reverse: CGGAGGTCCGTCGGGAGACATT |
| **PAK1** | Forward: CAGGACAGGAGGTGGCCATTAAGC  Reverse: CCACAGCTCATCTCCCACGAGG |
| **GFPT2** | Forward: CCTGCTCCTTGCCCATAGTAAA  Reverse: CCCACTTGAAACTACTCTCTTGCA |
| **GUSB** | Forward: CCAAAAAGTGCAGCGTTCCT  Reverse: ACCTGGTTTCATTGGCAATCTT |
| **ACTB** | Forward: ACCGAGCGCGGCTACA  Reverse: CTTAATGTCACGCACGATTTCC |
| **HPRT-1** | Forward: CGTCTTGCTCGAGATGTGATG  Reverse: GAGCACACAGAGGGCTACAATG |
