# Supplementary material for: RNA-Seq of Human Breast Ductal Carcinoma In Situ Models Reveals Aldehyde Dehydrogenase Isoform 5A1 as a Novel Potential Target
Source: PLoS One. 2012 Dec 6;7(12):e50249. doi: 10.1371/journal.pone.0050249 (PMC3516505; doi:10.1371/journal.pone.0050249)
Supplement: Table S2 — Generation of clusters from the reads obtained by deep sequencing of different samples. Biological duplicates of MCF10A, MCF10.DCIS, SUM102 and SUM225 samples were run in the Solexa flowcell. No. of reads indicates the total number of short reads that uniquely aligned to reference genome. The reads from each sample were grouped into clusters using two parameters: 1. window size 100 bp; 2. number of reads per cluster ≥9. No. of clusters indicates those generated from the reads based on Poisson distribution. The reads that did not group in any cluster were considered as background and discarded. Clusters/percentage indicates reads in clusters compared with the total number of reads. (DOC) [file pone.0050249.s006.doc]

**Table S2**

| **Sample** | **No. of reads** | **No. of clusters** | **Clusters/percentage** |
| --- | --- | --- | --- |
| MCF10A-2 | 10,465,483 | 97,237 | 8,952,978 (84.6%) |
| MCF10A-3 | 9,158,711 | 80,872 | 7,920,391 (86.5%) |
| DCIS-2 | 9,576,827 | 86,842 | 7,904,905 (82.5%) |
| DCIS-3 | 7,566,009 | 82,166 | 6,390,646 (84.5%) |
| SUM102-1 | 10,052,168 | 87,122 | 8,531,282 (84.9%) |
| SUM102-2 | 6,968,895 | 74,876 | 5,706,991 (81.9%) |
| SUM225-1 | 9,906,081 | 92,650 | 8,404,428 (84.8%) |
| SUM225-2 | 8,337,795 | 84,549 | 6,866,336 (82.4%) |
