# Supplementary material for: RNA-Seq of Human Breast Ductal Carcinoma In Situ Models Reveals Aldehyde Dehydrogenase Isoform 5A1 as a Novel Potential Target
Source: PLoS One. 2012 Dec 6;7(12):e50249. doi: 10.1371/journal.pone.0050249 (PMC3516505; doi:10.1371/journal.pone.0050249)
Supplement: Table S3 — Significantly differentially expressed genes common to all three DCIS models (MCF10.DCIS, SUM102 and SUM225) in comparison to MCF10A. The values in each column represent the log2 fold change in a DCIS model over MCF10A with adjusted p-value. “Inf” indicates that no reads mapped to that DCIS model. (DOC) [file pone.0050249.s007.doc]

**Table** S3

|  |  | **DCIS_vs_MCF10A** | | **SUM102_vs_MCF10A** | | **SUM225_vs_MCF10A** | |
| --- | --- | --- | --- | --- | --- | --- | --- |
| GeneID | **Symbol** | **log2Fold Change** | **padj** | **log2Fold Change** | **padj** | **log2Fold Change** | **padj** |
| 12 | SERPINA3 | 2.441 | 0.000 | -3.206 | 0.000 | -4.922 | 0.000 |
| 125 | ADH1B | -Inf | 0.000 | -Inf | 0.000 | -Inf | 0.000 |
| 247 | ALOX15B | -3.562 | 0.000 | -4.179 | 0.000 | 2.060 | 0.000 |
| 250 | ALPP | -2.829 | 0.000 | 3.087 | 0.000 | -2.956 | 0.000 |
| 347 | APOD | 2.772 | 0.000 | 5.521 | 0.000 | 4.424 | 0.000 |
| 348 | APOE | -7.323 | 0.000 | -3.308 | 0.000 | -6.379 | 0.000 |
| 443 | ASPA | -4.969 | 0.000 | -Inf | 0.000 | -Inf | 0.000 |
| 633 | BGN | -6.069 | 0.000 | -8.027 | 0.000 | -5.054 | 0.000 |
| 667 | DST | -2.945 | 0.000 | -3.362 | 0.000 | -2.407 | 0.000 |
| 715 | C1R | -4.216 | 0.000 | -2.270 | 0.000 | -6.984 | 0.000 |
| 768 | CA9 | 3.369 | 0.000 | -4.963 | 0.000 | -4.577 | 0.000 |
| 810 | CALML3 | -8.707 | 0.000 | -3.321 | 0.000 | -7.863 | 0.000 |
| 827 | CAPN6 | 3.035 | 0.000 | -7.579 | 0.000 | -Inf | 0.000 |
| 924 | CD7 | -4.762 | 0.000 | -5.703 | 0.000 | -3.582 | 0.000 |
| 970 | CD70 | -2.313 | 0.000 | -3.469 | 0.000 | -5.589 | 0.000 |
| 972 | CD74 | 2.375 | 0.000 | -3.512 | 0.000 | 4.732 | 0.000 |
| 1012 | CDH13 | -7.113 | 0.000 | -3.180 | 0.000 | -8.836 | 0.000 |
| 1131 | CHRM3 | 3.020 | 0.000 | -Inf | 0.000 | -3.503 | 0.000 |
| 1277 | COL1A1 | -4.160 | 0.000 | -2.486 | 0.000 | -3.065 | 0.000 |
| 1280 | COL2A1 | -5.783 | 0.000 | -3.170 | 0.000 | -Inf | 0.000 |
| 1288 | COL4A6 | -3.427 | 0.000 | -2.433 | 0.000 | -7.475 | 0.000 |
| 1294 | COL7A1 | -2.576 | 0.000 | -2.491 | 0.000 | -5.730 | 0.000 |
| 1295 | COL8A1 | 3.055 | 0.000 | -2.052 | 0.000 | -Inf | 0.000 |
| 1308 | COL17A1 | -3.105 | 0.000 | -4.103 | 0.000 | -10.474 | 0.000 |
| 1356 | CP | 3.314 | 0.000 | -4.829 | 0.000 | -4.437 | 0.000 |
| 1363 | CPE | 2.297 | 0.000 | 2.816 | 0.000 | 4.336 | 0.000 |
| 1364 | CLDN4 | 2.193 | 0.000 | 5.508 | 0.000 | 4.209 | 0.000 |
| 1435 | CSF1 | -3.621 | 0.000 | -2.563 | 0.000 | -4.493 | 0.000 |
| 1440 | CSF3 | -4.240 | 0.000 | 2.743 | 0.000 | -3.372 | 0.000 |
| 1634 | DCN | -6.528 | 0.000 | -5.016 | 0.000 | -Inf | 0.000 |
| 1796 | DOK1 | 2.379 | 0.000 | -3.284 | 0.000 | -4.355 | 0.000 |
| 1805 | DPT | -4.173 | 0.001 | -Inf | 0.000 | -Inf | 0.000 |
| 1836 | SLC26A2 | 2.359 | 0.000 | -3.389 | 0.000 | -5.882 | 0.000 |
| 1907 | EDN2 | -2.138 | 0.000 | -4.704 | 0.000 | -4.482 | 0.000 |
| 1999 | ELF3 | 2.444 | 0.000 | 4.181 | 0.000 | 2.364 | 0.000 |
| 2239 | GPC4 | 2.578 | 0.000 | -2.522 | 0.000 | -Inf | 0.000 |
| 2261 | FGFR3 | -7.542 | 0.000 | -3.606 | 0.000 | -2.700 | 0.000 |
| 2281 | FKBP1B | -3.561 | 0.000 | -3.721 | 0.000 | -Inf | 0.000 |
| 2308 | FOXO1 | -2.927 | 0.000 | -2.732 | 0.000 | -3.423 | 0.000 |
| 2525 | FUT3 | 2.889 | 0.000 | 4.143 | 0.000 | 4.379 | 0.000 |
| 2532 | DARC | -Inf | 0.000 | -5.146 | 0.000 | -Inf | 0.000 |
| 2535 | FZD2 | -2.428 | 0.000 | -2.542 | 0.000 | -2.804 | 0.000 |
| 2628 | GATM | 3.253 | 0.000 | -Inf | 0.000 | 5.273 | 0.000 |
| 2706 | GJB2 | 3.192 | 0.000 | 4.106 | 0.000 | -2.180 | 0.000 |
| 2737 | GLI3 | -3.090 | 0.000 | -2.058 | 0.000 | -3.839 | 0.000 |
| 2741 | GLRA1 | -3.123 | 0.000 | -4.439 | 0.000 | -2.372 | 0.000 |
| 3092 | HIP1 | -3.918 | 0.000 | -2.218 | 0.000 | -3.897 | 0.000 |
| 3176 | HNMT | 2.763 | 0.000 | 2.172 | 0.001 | 3.821 | 0.000 |
| 3204 | HOXA7 | 3.178 | 0.000 | 3.372 | 0.000 | 2.373 | 0.000 |
| 3382 | ICA1 | 2.135 | 0.000 | 2.625 | 0.000 | 4.032 | 0.000 |
| 3399 | ID3 | -3.951 | 0.000 | -2.384 | 0.000 | -2.785 | 0.000 |
| 3492 | IGH | -Inf | 0.000 | -Inf | 0.000 | -2.723 | 0.000 |
| 3635 | INPP5D | -3.269 | 0.000 | -2.288 | 0.000 | -5.240 | 0.000 |
| 3691 | ITGB4 | 2.208 | 0.000 | -3.660 | 0.000 | -6.051 | 0.000 |
| 3745 | KCNB1 | -2.651 | 0.000 | -5.289 | 0.000 | -5.904 | 0.000 |
| 3853 | KRT6A | -2.053 | 0.000 | 2.360 | 0.000 | -4.099 | 0.000 |
| 3854 | KRT6B | 2.823 | 0.000 | 2.275 | 0.000 | -3.748 | 0.000 |
| 3855 | KRT7 | 2.694 | 0.000 | 4.091 | 0.000 | 3.736 | 0.000 |
| 3861 | KRT14 | -7.433 | 0.000 | -2.679 | 0.000 | -9.086 | 0.000 |
| 3912 | LAMB1 | -3.809 | 0.000 | -3.968 | 0.000 | -7.238 | 0.000 |
| 3934 | LCN2 | 2.234 | 0.000 | 6.333 | 0.000 | 3.043 | 0.000 |
| 4017 | LOXL2 | 3.013 | 0.000 | -3.312 | 0.000 | -3.514 | 0.000 |
| 4064 | CD180 | 2.852 | 0.000 | -Inf | 0.001 | -Inf | 0.000 |
| 4129 | MAOB | 2.017 | 0.000 | -2.860 | 0.000 | -Inf | 0.000 |
| 4241 | MFI2 | 2.698 | 0.000 | -4.864 | 0.000 | -4.476 | 0.000 |
| 4313 | MMP2 | 2.826 | 0.000 | -5.679 | 0.000 | -5.295 | 0.000 |
| 4493 | MT1E | -6.242 | 0.000 | -3.778 | 0.000 | -8.621 | 0.000 |
| 4684 | NCAM1 | 2.979 | 0.000 | -3.705 | 0.000 | -Inf | 0.000 |
| 4776 | NFATC4 | 2.660 | 0.000 | 3.424 | 0.000 | 3.346 | 0.000 |
| 4811 | NID1 | -Inf | 0.000 | -6.183 | 0.000 | -Inf | 0.000 |
| 4915 | NTRK2 | 2.726 | 0.000 | -Inf | 0.000 | -Inf | 0.000 |
| 5063 | PAK3 | 2.061 | 0.000 | -Inf | 0.000 | -3.541 | 0.000 |
| 5067 | CNTN3 | 3.073 | 0.000 | -2.530 | 0.001 | -Inf | 0.000 |
| 5099 | PCDH7 | -5.215 | 0.000 | -2.127 | 0.000 | -2.353 | 0.000 |
| 5137 | PDE1C | 2.669 | 0.000 | -Inf | 0.000 | -Inf | 0.000 |
| 5155 | PDGFB | 2.412 | 0.000 | 2.162 | 0.000 | 3.569 | 0.000 |
| 5176 | SERPINF1 | -3.726 | 0.000 | -2.540 | 0.000 | -5.328 | 0.000 |
| 5376 | PMP22 | 2.942 | 0.000 | -2.898 | 0.000 | -Inf | 0.000 |
| 5603 | MAPK13 | 2.001 | 0.000 | 2.580 | 0.000 | 3.463 | 0.000 |
| 5654 | HTRA1 | -3.437 | 0.000 | -3.719 | 0.000 | -3.027 | 0.000 |
| 5909 | RAP1GAP | 2.261 | 0.000 | 2.015 | 0.000 | 6.110 | 0.000 |
| 5990 | RFX2 | -4.368 | 0.000 | -2.052 | 0.000 | -2.083 | 0.000 |
| 5997 | RGS2 | -2.757 | 0.000 | 3.436 | 0.000 | -3.013 | 0.000 |
| 6090 | RNY5 | -Inf | 0.000 | -Inf | 0.000 | -2.742 | 0.000 |
| 6271 | S100A1 | 2.899 | 0.000 | -4.376 | 0.000 | -2.759 | 0.000 |
| 6286 | S100P | 2.435 | 0.000 | 4.158 | 0.000 | 2.803 | 0.000 |
| 6319 | SCD | 2.367 | 0.000 | 2.063 | 0.000 | 3.612 | 0.000 |
| 6364 | CCL20 | 3.329 | 0.000 | 5.673 | 0.000 | 3.656 | 0.000 |
| 6376 | CX3CL1 | 2.963 | 0.000 | -4.851 | 0.000 | 6.709 | 0.000 |
| 6398 | SECTM1 | -4.361 | 0.000 | -3.122 | 0.000 | -4.039 | 0.000 |
| 6441 | SFTPD | -2.912 | 0.000 | -2.182 | 0.001 | -2.379 | 0.000 |
| 6542 | SLC7A2 | -Inf | 0.000 | -2.339 | 0.000 | -4.542 | 0.000 |
| 6947 | TCN1 | 2.468 | 0.000 | -2.744 | 0.000 | -4.258 | 0.000 |
| 7045 | TGFBI | -5.095 | 0.000 | -6.042 | 0.000 | -11.619 | 0.000 |
| 7049 | TGFBR3 | -3.143 | 0.000 | -6.830 | 0.000 | -4.613 | 0.000 |
| 7078 | TIMP3 | -4.420 | 0.000 | -3.086 | 0.000 | -5.973 | 0.000 |
| 7092 | TLL1 | -Inf | 0.000 | -3.983 | 0.000 | -Inf | 0.000 |
| 7097 | TLR2 | -4.569 | 0.000 | 3.708 | 0.000 | 2.803 | 0.000 |
| 7161 | TP73 | -4.437 | 0.000 | -4.972 | 0.000 | -2.587 | 0.000 |
| 7291 | TWIST1 | -2.128 | 0.000 | -2.631 | 0.000 | -8.800 | 0.000 |
| 7368 | UGT8 | 2.705 | 0.000 | -Inf | 0.000 | -3.861 | 0.000 |
| 7570 | ZNF22 | 2.191 | 0.000 | 2.647 | 0.000 | 3.397 | 0.000 |
| 7704 | ZBTB16 | -7.287 | 0.000 | -4.061 | 0.000 | -4.390 | 0.000 |
| 7915 | ALDH5A1 | 2.146 | 0.000 | 2.688 | 0.000 | 2.968 | 0.000 |
| 8343 | HIST1H2BF | 2.711 | 0.000 | 3.648 | 0.000 | 3.230 | 0.000 |
| 8515 | ITGA10 | 2.571 | 0.000 | -4.867 | 0.000 | -5.428 | 0.000 |
| 8645 | KCNK5 | 2.642 | 0.000 | -2.994 | 0.000 | -2.380 | 0.000 |
| 8698 | S1PR4 | -2.036 | 0.000 | -5.305 | 0.000 | -Inf | 0.000 |
| 8706 | B3GALNT1 | 2.925 | 0.000 | 2.626 | 0.000 | 4.365 | 0.000 |
| 8839 | WISP2 | -6.739 | 0.000 | -6.653 | 0.000 | -3.848 | 0.000 |
| 8991 | SELENBP1 | -2.130 | 0.000 | -2.666 | 0.000 | 2.362 | 0.000 |
| 9080 | CLDN9 | 2.809 | 0.001 | 3.066 | 0.000 | 4.958 | 0.000 |
| 9227 | LRAT | 3.034 | 0.000 | -3.331 | 0.000 | -2.678 | 0.000 |
| 9241 | NOG | 2.033 | 0.000 | -2.739 | 0.000 | -2.349 | 0.000 |
| 9289 | GPR56 | 2.687 | 0.000 | 2.396 | 0.000 | 3.387 | 0.000 |
| 9435 | CHST2 | -3.568 | 0.000 | -2.323 | 0.000 | -4.735 | 0.000 |
| 9586 | CREB5 | 3.117 | 0.000 | 4.440 | 0.000 | 3.586 | 0.000 |
| 9610 | RIN1 | -2.134 | 0.000 | -2.229 | 0.000 | -5.315 | 0.000 |
| 9651 | PLCH2 | -4.224 | 0.000 | -3.027 | 0.000 | -5.950 | 0.000 |
| 9672 | SDC3 | -2.707 | 0.000 | -5.151 | 0.000 | -2.433 | 0.000 |
| 9750 | FAM65B | -5.709 | 0.000 | -2.481 | 0.000 | -3.851 | 0.000 |
| 9805 | SCRN1 | 2.248 | 0.000 | 3.101 | 0.000 | 2.684 | 0.000 |
| 9839 | ZEB2 | -3.903 | 0.000 | -3.420 | 0.000 | -4.028 | 0.000 |
| 9843 | HEPH | 2.931 | 0.000 | -3.106 | 0.000 | -4.304 | 0.000 |
| 9976 | CLEC2B | -2.019 | 0.000 | -2.691 | 0.000 | -3.807 | 0.000 |
| 10170 | DHRS9 | 2.139 | 0.000 | 2.184 | 0.000 | -3.197 | 0.000 |
| 10232 | MSLN | -6.636 | 0.000 | -4.824 | 0.000 | -6.784 | 0.000 |
| 10388 | SYCP2 | 2.147 | 0.000 | -3.067 | 0.000 | 3.298 | 0.000 |
| 10398 | MYL9 | -3.996 | 0.000 | -7.321 | 0.000 | -3.871 | 0.000 |
| 10409 | BASP1 | -3.345 | 0.000 | 3.669 | 0.000 | -2.612 | 0.000 |
| 10544 | PROCR | -2.226 | 0.000 | -3.217 | 0.000 | -7.960 | 0.000 |
| 10551 | AGR2 | 3.043 | 0.000 | 5.173 | 0.000 | 6.818 | 0.000 |
| 10630 | PDPN | -Inf | 0.000 | -3.270 | 0.000 | -Inf | 0.000 |
| 10631 | POSTN | -4.984 | 0.000 | -4.897 | 0.000 | -Inf | 0.000 |
| 10656 | KHDRBS3 | 2.442 | 0.000 | -6.158 | 0.000 | -2.654 | 0.000 |
| 10816 | SPINT3 | -Inf | 0.000 | -Inf | 0.000 | -Inf | 0.000 |
| 10942 | PRSS21 | 2.541 | 0.000 | -2.337 | 0.000 | -2.983 | 0.000 |
| 11341 | SCRG1 | 3.036 | 0.000 | -Inf | 0.000 | -Inf | 0.000 |
| 22997 | IGSF9B | -3.340 | 0.000 | -7.496 | 0.000 | -7.112 | 0.000 |
| 23043 | TNIK | -2.178 | 0.000 | -3.511 | 0.000 | -3.899 | 0.000 |
| 23087 | TRIM35 | -2.654 | 0.000 | -2.311 | 0.000 | -4.489 | 0.000 |
| 23105 | FSTL4 | -Inf | 0.000 | -Inf | 0.000 | -2.512 | 0.001 |
| 23157 | SEPT6 | 2.686 | 0.000 | 3.911 | 0.000 | 4.444 | 0.000 |
| 23255 | KIAA0802 | -2.025 | 0.000 | -2.107 | 0.000 | -2.305 | 0.000 |
| 23284 | LPHN3 | 2.581 | 0.000 | -Inf | 0.000 | -4.298 | 0.000 |
| 23329 | TBC1D30 | 2.240 | 0.000 | 2.995 | 0.000 | 3.613 | 0.000 |
| 23507 | LRRC8B | 2.530 | 0.000 | 2.139 | 0.001 | 3.515 | 0.000 |
| 23555 | TSPAN15 | 2.187 | 0.000 | 2.668 | 0.000 | 4.121 | 0.000 |
| 23767 | FLRT3 | -2.666 | 0.000 | -3.197 | 0.000 | -4.825 | 0.000 |
| 25791 | NGEF | 2.779 | 0.000 | -2.984 | 0.000 | -2.309 | 0.000 |
| 25841 | ABTB2 | 2.019 | 0.000 | 2.541 | 0.000 | 2.685 | 0.000 |
| 26018 | LRIG1 | 2.309 | 0.000 | -2.865 | 0.000 | 2.113 | 0.000 |
| 26050 | SLITRK5 | -4.489 | 0.000 | -Inf | 0.000 | -Inf | 0.000 |
| 26227 | PHGDH | 2.435 | 0.000 | 2.410 | 0.000 | 5.083 | 0.000 |
| 26287 | ANKRD2 | -3.547 | 0.000 | -4.160 | 0.000 | -Inf | 0.000 |
| 26577 | PCOLCE2 | 2.816 | 0.000 | -5.106 | 0.000 | -Inf | 0.000 |
| 26579 | MYEOV | 3.023 | 0.000 | 2.768 | 0.000 | -3.461 | 0.000 |
| 26771 | SNORD102 | -Inf | 0.000 | -Inf | 0.000 | -Inf | 0.000 |
| 27065 | D4S234E | -2.525 | 0.000 | -3.951 | 0.000 | -6.959 | 0.000 |
| 27134 | TJP3 | 2.058 | 0.000 | 3.298 | 0.000 | 2.751 | 0.000 |
| 27293 | SMPDL3B | 2.346 | 0.000 | 2.777 | 0.000 | 3.074 | 0.000 |
| 28513 | CDH19 | 3.124 | 0.000 | -Inf | 0.000 | -Inf | 0.000 |
| 28999 | KLF15 | -2.775 | 0.000 | -Inf | 0.000 | -6.105 | 0.000 |
| 29841 | GRHL1 | 2.110 | 0.000 | 4.940 | 0.000 | 2.827 | 0.000 |
| 51170 | HSD17B11 | -3.063 | 0.000 | -2.196 | 0.000 | -Inf | 0.000 |
| 51330 | TNFRSF12A | 2.027 | 0.000 | 2.502 | 0.000 | 2.144 | 0.000 |
| 53947 | A4GALT | -4.483 | 0.000 | -8.590 | 0.000 | -8.787 | 0.000 |
| 54437 | SEMA5B | -5.613 | 0.000 | -3.554 | 0.000 | -Inf | 0.000 |
| 54453 | RIN2 | -2.088 | 0.000 | -2.021 | 0.000 | -2.507 | 0.000 |
| 54504 | CPVL | 2.730 | 0.000 | -2.780 | 0.000 | -4.335 | 0.000 |
| 54831 | BEST2 | -4.024 | 0.000 | -3.540 | 0.000 | -4.737 | 0.000 |
| 54836 | BSPRY | 3.081 | 0.000 | 3.141 | 0.000 | 3.034 | 0.000 |
| 54845 | ESRP1 | 2.763 | 0.000 | 3.781 | 0.000 | 3.845 | 0.000 |
| 54863 | C9orf167 | -3.087 | 0.000 | 2.709 | 0.000 | -2.732 | 0.000 |
| 55224 | ETNK2 | -5.539 | 0.000 | -4.243 | 0.000 | -3.650 | 0.000 |
| 55349 | CHDH | 2.685 | 0.000 | -3.598 | 0.000 | 3.350 | 0.000 |
| 55653 | BCAS4 | -2.511 | 0.000 | -2.115 | 0.000 | -3.019 | 0.000 |
| 56125 | PCDHB11 | -2.292 | 0.000 | -4.940 | 0.000 | -Inf | 0.000 |
| 56241 | SUSD2 | 2.512 | 0.000 | -2.093 | 0.000 | 3.972 | 0.000 |
| 57111 | RAB25 | 2.966 | 0.000 | 2.620 | 0.000 | 2.220 | 0.000 |
| 57484 | RNF150 | 2.770 | 0.000 | -4.221 | 0.000 | -Inf | 0.000 |
| 57604 | C8orf79 | -4.266 | 0.000 | -4.672 | 0.000 | -4.877 | 0.000 |
| 57639 | CCDC146 | -2.406 | 0.000 | -2.355 | 0.000 | -2.380 | 0.000 |
| 57863 | CADM3 | -7.274 | 0.000 | -7.246 | 0.000 | -Inf | 0.000 |
| 58473 | PLEKHB1 | 2.936 | 0.000 | -3.621 | 0.000 | -2.973 | 0.000 |
| 64063 | PRSS22 | 2.451 | 0.000 | 4.864 | 0.000 | 3.057 | 0.000 |
| 64218 | SEMA4A | 2.161 | 0.000 | 2.385 | 0.000 | 4.289 | 0.000 |
| 65987 | KCTD14 | 2.569 | 0.000 | -4.803 | 0.000 | -3.007 | 0.000 |
| 65989 | DLK2 | -3.014 | 0.000 | -5.486 | 0.000 | -4.097 | 0.000 |
| 65997 | RASL11B | -6.031 | 0.000 | -3.651 | 0.000 | -5.573 | 0.000 |
| 66002 | CYP4F12 | -6.324 | 0.000 | -3.236 | 0.000 | -3.627 | 0.000 |
| 78995 | C17orf53 | -2.335 | 0.000 | -2.662 | 0.000 | -2.094 | 0.000 |
| 79094 | CHAC1 | 2.283 | 0.000 | 5.749 | 0.000 | 4.450 | 0.000 |
| 79148 | MMP28 | -3.160 | 0.000 | -2.342 | 0.000 | -8.417 | 0.000 |
| 79156 | PLEKHF1 | 2.223 | 0.000 | 2.515 | 0.000 | 3.162 | 0.000 |
| 79603 | LASS4 | -Inf | 0.000 | -2.613 | 0.000 | 2.624 | 0.000 |
| 79783 | C7orf10 | -2.955 | 0.000 | -2.164 | 0.000 | -6.571 | 0.000 |
| 79919 | C2orf54 | 2.445 | 0.000 | 5.541 | 0.000 | 4.158 | 0.000 |
| 79977 | GRHL2 | 3.063 | 0.000 | 4.669 | 0.000 | 5.573 | 0.000 |
| 80023 | NRSN2 | 2.600 | 0.000 | -4.542 | 0.000 | 3.114 | 0.000 |
| 80117 | ARL14 | -5.054 | 0.000 | -3.440 | 0.000 | -4.234 | 0.000 |
| 80176 | SPSB1 | -2.947 | 0.000 | -2.522 | 0.000 | -2.585 | 0.000 |
| 81606 | LBH | -Inf | 0.000 | -2.638 | 0.000 | -4.530 | 0.000 |
| 81623 | DEFB126 | -Inf | 0.000 | -Inf | 0.000 | -Inf | 0.000 |
| 81848 | SPRY4 | 2.789 | 0.000 | 3.017 | 0.000 | 4.688 | 0.000 |
| 83700 | JAM3 | -4.476 | 0.000 | -5.389 | 0.000 | -3.996 | 0.000 |
| 84109 | QRFPR | 2.464 | 0.000 | -3.506 | 0.000 | -Inf | 0.000 |
| 84707 | BEX2 | 2.804 | 0.000 | 2.424 | 0.000 | 4.008 | 0.000 |
| 84913 | ATOH8 | -6.495 | 0.000 | -Inf | 0.000 | -2.271 | 0.000 |
| 84940 | CORO6 | -4.903 | 0.000 | -2.513 | 0.000 | -5.036 | 0.000 |
| 84969 | TOX2 | -4.599 | 0.000 | -5.204 | 0.000 | -Inf | 0.000 |
| 89932 | PAPLN | -4.569 | 0.000 | -3.066 | 0.000 | -3.549 | 0.000 |
| 90226 | UCN2 | -3.376 | 0.000 | -3.025 | 0.000 | -Inf | 0.000 |
| 90362 | FAM110B | -Inf | 0.000 | -4.142 | 0.001 | -4.328 | 0.000 |
| 90525 | SHF | 2.716 | 0.000 | -4.094 | 0.000 | -4.007 | 0.000 |
| 92126 | DSEL | -3.212 | 0.000 | -4.991 | 0.000 | -6.511 | 0.000 |
| 94162 | SNORD38A | -Inf | 0.000 | -Inf | 0.000 | -Inf | 0.000 |
| 114088 | TRIM9 | 2.939 | 0.000 | -Inf | 0.000 | -Inf | 0.000 |
| 114783 | LMTK3 | 2.533 | 0.000 | 2.532 | 0.000 | 4.652 | 0.000 |
| 114897 | C1QTNF1 | 2.579 | 0.000 | 2.253 | 0.000 | -4.141 | 0.000 |
| 117195 | MRGPRX3 | 2.793 | 0.000 | 3.479 | 0.000 | -4.783 | 0.000 |
| 117248 | GALNTL2 | -3.208 | 0.000 | -3.031 | 0.000 | -6.024 | 0.000 |
| 120892 | LRRK2 | -2.211 | 0.000 | -2.310 | 0.000 | -2.872 | 0.000 |
| 124930 | ANKRD13B | -2.138 | 0.000 | -2.878 | 0.000 | -4.047 | 0.000 |
| 124975 | GGT6 | 2.480 | 0.000 | 3.418 | 0.000 | 3.922 | 0.000 |
| 126433 | FBXO27 | 3.614 | 0.000 | 3.573 | 0.000 | 3.765 | 0.000 |
| 128218 | TMEM125 | 2.468 | 0.000 | 2.061 | 0.000 | 3.430 | 0.000 |
| 130497 | OSR1 | 2.896 | 0.000 | -5.165 | 0.000 | -6.362 | 0.000 |
| 135398 | C6orf141 | -2.378 | 0.000 | -2.487 | 0.000 | -5.232 | 0.000 |
| 144195 | SLC2A14 | -3.762 | 0.000 | -4.705 | 0.000 | -Inf | 0.000 |
| 144501 | KRT80 | 2.732 | 0.000 | 5.486 | 0.000 | 3.144 | 0.000 |
| 146439 | CCDC64B | 2.103 | 0.000 | 4.315 | 0.000 | 4.032 | 0.000 |
| 146802 | SLC47A2 | -4.069 | 0.000 | -6.340 | 0.000 | -4.536 | 0.000 |
| 147920 | IGFL2 | -Inf | 0.000 | -2.562 | 0.000 | -Inf | 0.000 |
| 151473 | SLC16A14 | 3.631 | 0.000 | 4.073 | 0.000 | 6.321 | 0.000 |
| 153346 | LOC153346 | -2.139 | 0.000 | -3.796 | 0.000 | -2.380 | 0.000 |
| 161291 | TMEM30B | 2.547 | 0.000 | 2.036 | 0.000 | 4.281 | 0.000 |
| 163259 | DENND2C | -2.984 | 0.000 | 2.142 | 0.000 | -3.336 | 0.000 |
| 165545 | DQX1 | -2.443 | 0.000 | -3.583 | 0.000 | -5.055 | 0.000 |
| 200634 | KRTCAP3 | 2.046 | 0.000 | -4.032 | 0.000 | -4.426 | 0.000 |
| 200879 | LIPH | 2.122 | 0.000 | 4.346 | 0.000 | 5.099 | 0.000 |
| 221400 | TDRD6 | -2.529 | 0.000 | -5.067 | 0.000 | -5.275 | 0.000 |
| 223075 | CCDC129 | 2.874 | 0.000 | -Inf | 0.000 | -Inf | 0.000 |
| 255189 | PLA2G4F | -5.278 | 0.000 | -2.672 | 0.000 | -5.253 | 0.000 |
| 257000 | PLAC2 | -3.129 | 0.000 | 3.075 | 0.000 | 2.297 | 0.000 |
| 283174 | LOC283174 | -3.309 | 0.000 | -Inf | 0.000 | -8.498 | 0.000 |
| 284759 | SIRPB2 | -3.718 | 0.000 | -3.657 | 0.000 | -5.024 | 0.000 |
| 285195 | SLC9A9 | -5.110 | 0.000 | -2.987 | 0.000 | -7.565 | 0.000 |
| 285944 | LOC285944 | 2.970 | 0.000 | 3.405 | 0.000 | 2.302 | 0.000 |
| 286527 | TMSB15B | -3.789 | 0.000 | -4.523 | 0.000 | -Inf | 0.000 |
| 340359 | KLHL38 | -6.074 | 0.000 | -3.846 | 0.000 | -3.402 | 0.000 |
| 341640 | FREM2 | 2.984 | 0.000 | -Inf | 0.000 | -Inf | 0.000 |
| 388611 | CD164L2 | -4.286 | 0.000 | -2.803 | 0.000 | -3.415 | 0.000 |
| 400945 | FLJ41481 | -3.785 | 0.000 | -Inf | 0.000 | -3.513 | 0.000 |
| 401827 | MSLNL | -5.539 | 0.000 | -5.449 | 0.000 | -Inf | 0.000 |
| 441869 | hCG_20426 | -3.645 | 0.000 | -2.587 | 0.000 | -4.776 | 0.000 |
| 642587 | LOC642587 | -4.208 | 0.000 | -3.985 | 0.000 | -6.783 | 0.000 |
| 643965 | TMEM88B | -3.851 | 0.000 | -2.530 | 0.000 | -3.990 | 0.000 |
| 645027 | EVPLL | -5.270 | 0.000 | -5.183 | 0.000 | -3.378 | 0.000 |
| 646543 | LOC646543 | -2.493 | 0.000 | -Inf | 0.000 | -8.203 | 0.000 |
| 692111 | SNORD71 | -2.111 | 0.000 | -3.051 | 0.000 | -3.067 | 0.000 |
| 692198 | SNORD78 | -2.494 | 0.000 | -2.464 | 0.000 | -2.470 | 0.000 |
| 727936 | GLT8D4 | 2.031 | 0.000 | -2.941 | 0.000 | -Inf | 0.000 |
| 728196 | LOC728196 | -5.172 | 0.000 | -5.142 | 0.000 | -Inf | 0.000 |
| 100128031 | LOC100128031 | 2.076 | 0.000 | 5.421 | 0.000 | 4.188 | 0.000 |
| 100128186 | LOC100128186 | -Inf | 0.000 | -Inf | 0.000 | -Inf | 0.000 |
| 100128501 | LOC100128501 | -4.274 | 0.000 | -2.714 | 0.000 | -2.707 | 0.000 |
| 100128703 | LOC100128703 | 2.802 | 0.000 | -2.357 | 0.000 | -3.554 | 0.000 |
| 100128770 | LOC100128770 | 2.819 | 0.000 | 4.698 | 0.000 | 4.696 | 0.000 |
| 100128789 | LOC100128789 | 4.767 | 0.000 | 5.212 | 0.000 | 5.226 | 0.000 |
| 100129500 | LOC100129500 | -7.347 | 0.000 | -3.416 | 0.000 | -6.607 | 0.000 |
| 100130131 | LOC100130131 | -Inf | 0.000 | -2.984 | 0.000 | -3.168 | 0.000 |
| 100131825 | LOC100131825 | -Inf | 0.000 | -Inf | 0.000 | -Inf | 0.000 |
| 100132240 | LOC100132240 | 2.607 | 0.000 | -2.316 | 0.000 | -6.213 | 0.000 |
| 100192386 | FLJ16779 | -4.969 | 0.000 | -4.939 | 0.000 | -Inf | 0.000 |
| 100286923 | LOC100286923 | -Inf | 0.000 | -4.482 | 0.000 | -3.546 | 0.000 |
| 100287089 | LOC100287089 | -3.047 | 0.000 | -2.669 | 0.000 | -6.761 | 0.000 |
| 100287407 | LOC100287407 | -2.154 | 0.000 | -3.512 | 0.000 | -5.293 | 0.000 |
| 100287558 | LOC100287558 | -Inf | 0.000 | -4.770 | 0.000 | -4.553 | 0.000 |
| 100288023 | LOC100288023 | -3.341 | 0.000 | -3.087 | 0.000 | -3.277 | 0.000 |
| 100288314 | LOC100288314 | 2.504 | 0.000 | 2.102 | 0.000 | 3.485 | 0.000 |
| 100288409 | LOC100288409 | -Inf | 0.000 | -2.860 | 0.000 | -2.382 | 0.000 |
| 100288670 | LOC100288670 | -2.681 | 0.000 | -5.780 | 0.000 | -2.276 | 0.000 |
| 100288776 | LOC100288776 | 2.615 | 0.000 | 3.669 | 0.000 | 3.610 | 0.000 |
| 100288925 | LOC100288925 | -Inf | 0.000 | -Inf | 0.000 | -6.036 | 0.000 |
| 100289165 | LOC100289165 | -5.790 | 0.000 | -4.591 | 0.000 | -4.152 | 0.000 |
| 100289344 | LOC100289344 | -3.842 | 0.000 | -Inf | 0.000 | -3.951 | 0.000 |
| 100289437 | LOC100289437 | -2.724 | 0.000 | -Inf | 0.000 | -5.107 | 0.000 |
| 100289488 | LOC100289488 | 2.465 | 0.000 | 4.144 | 0.000 | 2.317 | 0.000 |
| 100289567 | LOC100289567 | -3.524 | 0.000 | -2.549 | 0.000 | -4.655 | 0.000 |
| 100289603 | LOC100289603 | -3.837 | 0.000 | -Inf | 0.000 | -Inf | 0.000 |
| 100289660 | LOC100289660 | -4.173 | 0.001 | -Inf | 0.000 | -Inf | 0.000 |
| 100290920 | LOC100290920 | -5.694 | 0.000 | -6.189 | 0.000 | -Inf | 0.000 |
| 100293045 | LOC100293045 | -2.838 | 0.000 | -7.650 | 0.000 | -6.259 | 0.000 |
| 100294404 | LOC100294404 | -4.499 | 0.000 | -2.776 | 0.000 | -3.924 | 0.000 |
