# Supplementary material for: RNA-Seq of Human Breast Ductal Carcinoma In Situ Models Reveals Aldehyde Dehydrogenase Isoform 5A1 as a Novel Potential Target
Source: PLoS One. 2012 Dec 6;7(12):e50249. doi: 10.1371/journal.pone.0050249 (PMC3516505; doi:10.1371/journal.pone.0050249)
Supplement: Table S4 — Signaling pathway networks involving consistently differentially expressed genes. Ingenuity Pathway Analysis (IPA) of differentially expressed genes revealed several statistically significant pathway networks. Each network contains focus molecules that are obtained from RNA-Seq analysis and are indicated in bold. The other genes in the network are derived from IPA database. The statistical significance score and top functions associated with the molecules in network are also indicated in the table. (DOC) [file pone.0050249.s008.doc]

**Table S4**

| ID | Molecules in Network | Score | Focus Molecules | Top Functions |
| --- | --- | --- | --- | --- |
| 1 | 26s Proteasome,A4GALT,Akt,Alp,ANKRD2,Ap1,APOE,BEX2,BGN,C1q,C1R,CCL20,CD7,CD70,CDH13,CHEMOKINE,CLDN4,CLDN9,cldn,COL17A1,COL1A1,COL21A1,COL22A1,COL28A1,COL2A1,COL4A6,COL7A1,collagen,Collagen type I,Collagen type IV,Collagen(s),CPE,CSF1,Cyclin A,DARC,DCN,DST,EDN2,ELF3,ERK,ERK1/2,FGFR3,FKBP1B,FOXO1,FSH,FZD2,GLI3,Gpcr,GPR56,GPR61,GPR84,GPR113,GPR114,GPR144,GPR162,GPR174,GPR180,GPR89A/GPR89B,GPR89C,Growth hormone,HIST2H2BE,Histone h3,Histone h4,HOXA7,ID3,Ifn gamma,Ige,IgG1,IgG,IGH,Igm,IL1,INPP5D,Insulin,Integrin,Interferonalpha,Jnk,LAMB1,Laminin,LBH,LCN2,LDL,Lh,LMTK3, MAPK13,Mapk,MMP28,Mmp,MT1E,MYL9,NFAT (complex),Nfat (family),NFATC4,NFkB (complex),NID1,P38 MAPK,p85 (pik3r),PCDH7,Pdgf (complex),PDGF BB,PDGFB,PI3K (complex),Pkc(s),POSTN,PPARGC1B,PROCR,Rac,Rap1,RAP1GAP,Ras,RIN1,RIN2,S100P,S1PR4,SCD,SECTM1,SERPINF1,SFTPD,Smad,SPRY4,SPSB1,TAAR8,Tgf beta,TGFBI,TGFBR3,TIMP3,TJP3,TLL1,Tnf receptor,TNFRSF12A,TNIK,TP73,TWIST1,UCN2,Vegf,VN1R2,VN1R3,WISP2,ZBTB16ZEB2 | 131 | 74 | Connective Tissue Disorders, Genetic Disorder, Dermatological Diseases and Conditions |
| 2 | ACSL4,ACSM3,ADAMDEC1,ADCYAP1,AGR2,AMACR,ANKRD13B,ANP32E,APOD,arachidonic acid,ARL14,ART3,B3GALNT1,BCAS4,beta-estradiol,BSPRY,C9orf3,CADM1,CADM3,CCDC64B,CDK18,CHAC1,CKMT1A/CKMT1B,CLCN2,CLEC2B,Clec2d (includes others),CNN2,CORO6,CPXM1,CREB5,DLEU2,DLK2,DPT,DQX1,E2F1,Egfbp2,EGR2,ELOVL2,EPGN,ERBB2,FAM105B,FAM110B,FAM129A,FAM50A,FAM65B,FBXO27,FOS,FRRS1,FXYD5,G6pd2,GINS1,GLRA1,GLUD2,Gm10077,Gm10155,GRHL2,heparin,HIST1H2AG (includes others),HNMT,HTRA1,ICA1,IL6,IL29,INMT,JAM3,KDELR3,KIAA0802,KLK11,KRT7,KRT13,KRT14,KRT81,L-alpha-lysophosphatidylcholine, stearoyl,LEPROT,LIPH,LOC729505,LRRN3,LTBP3,MAP4,MAPK6,MARK4,MED31,MGMT,MIA2,MLXIP,MSGN1,MYC,OMG,PAQR7,PARD3,PCDHGC3,PDE7A,PDPN,PGLYRP1,PHGDH,PLA2G2E,PLA2G2F,PLA2G4F,PLSCR1,PMPCB,PPT2,progesterone,PRR15L,PRSS22,RAB10,RAB25,RFX2,RPL17,Rpl9 (includes others),RPLP1,RPS18,SATB1,SBNO2,SCRN1,SDC3,SEMA4A,SEPT6,SLC13A3,SLC14A1,SLC39A14,SLC7A2,SOD2,SOLH,SPAG4,SPEG,SPRR3,SQRDL,TDRD6,TERT,TGFB1,Timd2,TMEM2,TMEM126A,TOX2,TRIM14,UST,YWHAZ,YY2,ZFP161,ZNF22 | 65 | 43 | Cellular Development, Lipid Metabolism, Molecular Transport |
| 3 | AATK,ABCB9,ABTB2,ACY1,ACY3,ADCK3,ADH6,ADH1B,ALDH16A1,ALDH1L1,ALDH5A1,ALDH8A1,ANKRD49,APP,AQP9,AS3MT,ASPA,BCL2L14,BPI,C21orf33,C7orf10,C8orf4,CALML3,CALML4,Casein,CCDC76,CD300C,CDCA7L,CES2,CHST2,CHST4,CIDEC,COX11,CSRNP1,CTNNBL1,CWC15,CYP26B1,CYP4F3,D4S234E,DDX10,DDX18,DEFA4,DHRS3,DHRS4,DLEU1,ECD,ECE2,ECI2,EIF4EBP2,ELMOD3,EPB41L4B,ETNK2,EWSR1,FAM86C,FARSB,FASTKD2,FETUB,FEZF2,FLRT3,FUT3,FUT4,FZD3,GGT6,GPX2,GRHL1,HGD,HIP1,HMGN4,HNF4A,HS3ST1,HS6ST1,HSD17B11,IFT122,KCNB1,KCNQ5,KIF3C,KLF15,LAD1,LGALS12,LRRC8C,LRRK2,MAP7,MGST2,MMP28,MRPS18B,MSLN,MTF2,NBPF3,NDST1,NDUFB1,NDUFV1,NLN,NR3C1,NR4A2,OAS3,OASL,OSCAR,OTUD6B,PAFAH2,PARP4,PHB2,PLCH2,PLEKHF1,PWP1,RABGGTB,RASL11B,RNY5,RTP3,SAMHD1,SEMA3C,SEMA5B,SEPX1,SERPINB8,SLC25A20,SLC38A1,SLC5A3,STARD10,STT3A,SULT1C2,SUPT4H1,TMEM176B,TNF,TNFRSF21,TPCN1,TPP2,tretinoin,TRIM15,TRIM35,TROVE2,TSPAN14,VMP1,WDR37,WNT10A,WTAP,ZBTB11,ZDHHC6,ZNF133,ZNF146,ZNF318 | 32 | 26 | Lipid Metabolism, Small Molecule Biochemistry, Organismal Injury and Abnormalities |
