# Supplementary material for: RNA-Seq of Human Breast Ductal Carcinoma In Situ Models Reveals Aldehyde Dehydrogenase Isoform 5A1 as a Novel Potential Target
Source: PLoS One. 2012 Dec 6;7(12):e50249. doi: 10.1371/journal.pone.0050249 (PMC3516505; doi:10.1371/journal.pone.0050249)
Supplement: Table S5 — Expression levels of selected genes in three models of DCIS in comparison to normal human mammary epithelial cells (HMEC). The expression of all genes by qRT-PCR was normalized to that of β-glucuronidase (GUSB) as a housekeeping gene. The values in the table are Log2 (Fold Change), positive values indicate up-regulation in the DCIS model (highlighted in green); negative values indicate down-regulation (highlighted in red). (DOC) [file pone.0050249.s009.doc]

**Table S5**

| **Gene Symbol** | **DCIS** | **SUM102** | **SUM225** |
| --- | --- | --- | --- |
| **TIMP3** | 0.39 | 1.76 | -3.10 |
| **PAK1** | -4.55 | -2.79 | -4.39 |
| **S100P** | 8.27 | 11.01 | 9.26 |
| **IRS1** | -4.51 | -3.02 | -4.66 |
| **CASP2** | 0.98 | 1.13 | 0.68 |
| **MET** | -5.65 | -3.21 | -5.74 |
| **FOXO3** | -4.45 | -2.35 | -6.10 |
| **RHOB** | -3.46 | -2.22 | -0.30 |
| **DUSP5** | -4.39 | -2.24 | -2.82 |
| **ALDH5A1** | 2.39 | 2.99 | 2.98 |
| **GLUD1** | -1.25 | -0.61 | -0.73 |
| **GLUL** | -3.61 | -5.14 | -3.44 |
| **GFPT2** | -0.88 | 2.72 | 0.87 |
